# Peroxisomes support human herpesvirus 8 latency by stabilizing the viral oncogenic protein vFLIP via the MAVS-TRAF complex

**DOI:** 10.1371/journal.ppat.1007058

**Published:** 2018-05-10

**Authors:** Young Bong Choi, Yeeun Choi, Edward William Harhaj

**Affiliations:** 1 Department of Oncology, Sidney Kimmel Comprehensive Cancer Center, Johns Hopkins University School of Medicine, Baltimore, Maryland, United States of America; 2 Centennial High School, Ellicott City, Maryland, United States of America; University of Southern California, UNITED STATES

## Abstract

Human herpesvirus 8 (HHV-8) is causally related to human malignancies. HHV-8 latent viral FLICE-inhibitory protein (vFLIP) is a viral oncoprotein that is linked to pathogenesis, but how its expression is regulated is largely unknown. In an attempt to understand the role of the mitochondrial antiviral signaling (MAVS) adaptor in HHV-8 infection, we discovered that vFLIP expression was post-translationally up-regulated by the MAVS signaling complex on peroxisomes. Furthermore, we demonstrated that vFLIP could be targeted to the peroxisomes, where it was oncogenically active, in a PEX19-dependent manner. Targeted disruption of vFLIP and MAVS interaction resulted in a decrease in vFLIP expression and selectively promoted death of latently HHV-8-infected cells, providing therapeutic potential for treating HHV-8 diseases. Collectively, our experimental results suggest novel involvement of peroxisomes and MAVS in the stabilization of vFLIP and thereby in the establishment or maintenance of HHV-8 latency and associated pathogenesis.

## Author summary

HHV-8, also known as Kaposi’s sarcoma associated herpesvirus (KSHV), is the etiological agent of several malignancies occurring in individuals with severe immunosuppression, as occurs in acquired immunodeficiency syndrome and transplantation. The viral latent proteins, required for persistent infection, are believed to contribute to pathogenesis. Amongst them, vFLIP has an oncogenic potential through activation of NF-κB and suppression of cell death induced by apoptosis and autophagy. Despite its functional significance, how the stability of the protein is regulated remains elusive. Here, we found that vFLIP can localize to peroxisomes where it is stabilized by its association with the mitochondria antiviral signaling adaptor protein MAVS, which mediates K63-linked polyubiquitination of vFLIP by TNF receptor-associated factors (TRAFs) and renders it resistant to degradation induced by autophagy and the ubiquitin-proteasome pathway. In addition, our genetic studies using MAVS-deficient HHV-8-infected cells revealed that MAVS is essential for protection of HHV-8-infected cells from autophagic cell death via its stabilization of vFLIP. Furthermore, cell penetrating peptide-mediated disruption of the interaction between MAVS and vFLIP led to vFLIP degradation and death of HHV-8-infected tumor cells. Taken together, we have identified a novel proviral function of peroxisome-localized MAVS mediated through vFLIP stabilization and consequent promotion of HHV-8 latency.

## Introduction

In response to virus infection, a host innate immune response is activated to restrict virus replication and dissemination. Activation of innate immune antiviral responses relies on signaling pathways relaying viral nucleic acid recognition to the transcriptional machinery. Upon RNA virus infection, viral RNA is recognized by a class of specialized pattern recognition receptors (PRRs); cytosolic viral RNA is sensed by RIG-I-like receptors (RLRs) including RIG-I and MDA5 [1, 2], and viral RNA in endosomes is sensed by Toll-like receptors (TLRs) including TLR3 and TLR7 [3, 4]. Upon recognition of these RNA species, RLRs and TLRs recruit specific intracellular adaptor proteins to initiate signaling pathways culminating in activation of transcription factors, nuclear factor-κB (NF-κB) and interferon (IFN) regulatory factors (IRFs), that upregulate expression of antiviral cytokines. On the other hand, there is accumulating evidence that RIG-I has a role in the detection of several DNA viruses including adenovirus, herpes simplex virus 1, hepatitis B virus, Epstein-Barr virus (EBV), and human herpesvirus 8 (HHV-8, also called Kaposi sarcoma-associated herpesvirus (KSHV)) [5–9], in some cases by recognizing intermediate RNA species that are generated by RNA polymerase II or III [5, 10].

In the RLR signaling pathway, the mitochondrial protein MAVS (also known as IPS-1, CARDIF, and VISA) plays a pivotal role as an adaptor. Once activated by the viral RNA-RLR complex, MAVS polymerizes into prion-like filament structures [11], which nucleate the assembly of a large signalosome complex consisting of the tumor necrosis factor (TNF) receptor-associated factor (TRAF) proteins [12, 13], the TNF receptor type 1-associated death domain protein (TRADD) ternary complex [14], the IκB kinase (IKK) complex (IKKα, IKKβ, and IKKγ), and IKK-related kinases TBK1 and IKKε [15]. The MAVS signalosome promotes the activation of IRF3 and/or IRF7 and NF-κB, which then induce the transcriptional activation of type I IFN, proinflammatory cytokines, and a slew of IFN-stimulated genes (ISGs) to establish an antiviral milieu in infected and uninfected host cells. MAVS can also localize to peroxisomes where it initiates downstream signaling that induces rapid IFN-independent ISG expression, and preferential expression of type III IFN [16, 17], although there was a conflicting report that mitochondria-localized MAVS can induce a robust type III IFN response in virus-infected hepatoma cells [18]. In addition, MAVS has been implicated in mediating virus-induced apoptotic cell death. This antiviral activity of MAVS appears to be dependent on its localization to mitochondria rather than peroxisomes [19, 20], inducing mitochondrial dysfunction and oxidative stress by interacting with cellular proapoptotic effector proteins including SARM1 [21], VDAC1 [20], MKK7/JNK2 [22], and caspase-8 [23].

HHV-8 is the etiological agent of Kaposi sarcoma and two lymphoproliferative diseases, primary effusion lymphoma (PEL) and multicentric Castleman’s disease [24, 25]. Like other herpesviruses, HHV-8 exhibits two distinct phases of infection: latency and lytic (productive) replication. We and another group reported that MAVS may function as a negative regulator to suppress HHV-8 productive replication [7, 26]. However, the precise role of MAVS in HHV-8 infection remains unknown. In this study, we discovered a novel and essential function of MAVS in successful maintenance of HHV-8 latent infection by stabilizing HHV-8 viral FLICE-inhibitory protein (vFLIP) on peroxisomes. Furthermore, we propose that targeted modulation of the vFLIP-MAVS interaction may have therapeutic potential for treatment of HHV-8 diseases.

## Results

### MAVS is required for the survival of latently HHV-8-infected cells

**Fig 1:**
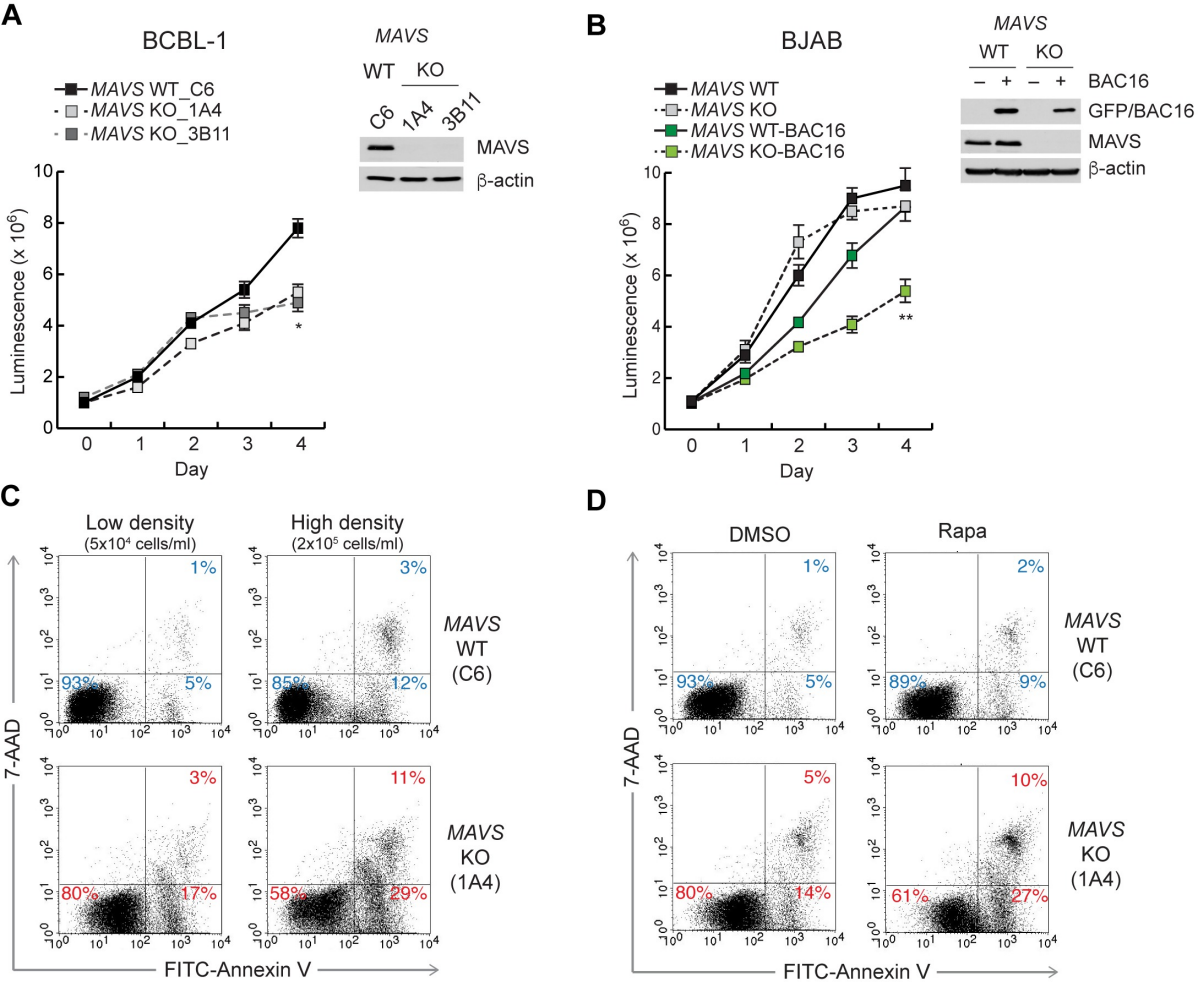
Increased death of HHV-8-infected MAVS-deficient cells. (A-B) Growth curve of wild-type (WT) and *MAVS* knockout (KO) BCBL-1 cell lines (A) and BJAB cells infected or uninfected with HHV-8 BAC16 virus (B). Cell viability was assessed by determining ATP levels using CellTiter-Glo for 4 days. MAVS and GFP expression were determined on day 0 by immunoblot as shown next to each graph. Data are presented as mean ± SD of triplicate samples. Probability (p) values at day 4 are depicted; * p<0.05 and ** p<0.01. (C-D) Flow cytometry analyses of annexin V-FITC and 7-AAD in WT and *MAVS* KO BCBL-1 (1A4) cells seeded at 5x10^4^ and 2x10^5^ cells/ml and incubated for 2 days (C) and seeded at 5x10^4^ cells/ml and treated with 20 nM rapamycin (Rapa) for 2 days (D). The percentage of total cells in each quadrant is shown.

To examine the effect of MAVS on HHV-8 infection, the expression of MAVS was genetically ablated by CRISPR/Cas9-mediated mutagenesis in BCBL-1, an HHV-8-infected PEL cell line, and control HHV-8-negative BJAB B-cell lymphoma cells. For subsequent studies, we used two *MAVS* knockout (KO) BCBL-1 cell lines (1A4 and 3B11) that were generated by two respective small guide RNAs (gRNAs) 1 and 3 (S1 Table), and two control cell lines (C3 and C6, hereafter referred to as wild-type (WT) BCBL-1 cells) that harbor empty transfer vector and Cas9. Consistent with a previous report [7], expression of lytic genes including MIR-2 (K5) and K8.1 was highly up-regulated in *MAVS* KO BCBL-1 cells after reactivation (S1A and S1B Fig). However, reactivation-induced expression of IFN-α and IFN-λ was not affected by MAVS deficiency in BCBL-1 cells (S1C and S1G Fig). Of note, IFN-β expression was not induced by reactivation in WT and *MAVS* KO BCBL-1 cells (S1E Fig). On the other hand, latent *MAVS* KO BCBL-1 cell lines displayed retarded growth under normal culture conditions (Fig 1A); however, growth of BJAB cells was not affected by MAVS deficiency (Fig 1B), suggesting that MAVS is necessary for proliferation of HHV-8-infected cells. To further test this idea, WT and *MAVS* KO BJAB cells were infected by the HHV-8 bacterial artificial chromosome 16 (BAC16) virus. The infection efficiency was comparable between the isogenic cell lines, as evidenced by green fluorescent protein (GFP) expression encoded by BAC16, albeit there was slightly less expression in the *MAVS* KO cells (Fig 1B). Notably, the growth of BAC16-infected *MAVS* KO BJAB cells was significantly retarded compared to control BJAB-BAC16 cells (Fig 1B).

Retarded cell growth could be an indication of cell death. Therefore, we examined whether the growth delay of HHV-8-infected *MAVS* KO cells was due to cell death. Cells were seeded at two different densities (5x10^4^ and 2x10^5^ cells/ml), cultured for 2 days, and analyzed by flow cytometry with FITC-annexin V and 7-AAD staining. While necrotic cells (annexin V^–^/7-AAD^+^) were barely detectable, early (annexin V^+^/7-AAD^–^) and late (annexin V^+^/7-AAD^+^) apoptotic populations increased about three-fold in both low- and high-density cultures of *MAVS* KO BCBL-1 cells compared to those of the WT cells (Fig 1C). High-density culture promoted an increase in the number of early and late apoptotic cells by at least two-fold compared to low-density culture (Fig 1C), resulting in a higher percentage of cell death in *MAVS* KO BCBL-1 (40%) than WT cells (15%). Interestingly, a pan caspase inhibitor, zVAD-fmk, only partially blocked early and late apoptosis in *MAVS* KO BCBL-1 cells (S2A Fig), suggesting that caspase-independent death may also be induced in *MAVS* KO HHV-8-infected cells. Moreover, it is conceivable that the increased death of *MAVS* KO BCBL-1 cells might be caused by enhanced virus replication, which is associated with increased apoptosis [27]. However, expression of lytic genes such as MIR-2 and K8.1 was not induced in latent *MAVS* KO BCBL-1 cells (S1A and S1B Fig) and there was no difference in spontaneous virus replication between *MAVS* WT and KO BCBL-1 cells under high-density culture conditions (S2B Fig).

In high-density culture, cells may readily undergo cell death due to deprivation of nutrients or the presence of toxic metabolites. Autophagy is a cytoprotective response to cellular stressors including nutrient and energy starvation [28], but it has also been implicated in a form of caspase-independent cell death, termed autophagic cell death [29] under certain conditions such as starvation [30, 31]. Thus, we hypothesized that HHV-8-infected *MAVS* KO cells cultured at high density may be susceptible to autophagic cell death, thus leading to retarded growth (Fig 1A). To examine this notion, the cells at low density were treated with rapamycin to induce autophagy. Indeed, rapamycin induced an increase in the percentage of annexin V-positive *MAVS* KO BCBL-1 cells to a similar degree as the high-density culture (Fig 1D; compare with Fig 1C). This result suggests that MAVS may be involved in the protection of HHV-8-infected cells from autophagic cell death. In addition, *MAVS* KO BCBL-1 cells rapidly lost their viability when incubated in Earle’s Balanced Salt Solution (EBSS), an autophagy inducer, for 6 h (S2C Fig). Intriguingly, the *MAVS* KO cells were also susceptible to death induced by TNF-related apoptosis-inducing ligand (TRAIL), but not by other cell death-inducing drugs including staurosporine and mitochondria-damaging drugs (S2C Fig). These results indicate that MAVS may confer selective protection to HHV-8-infected cells against death induced by autophagy and death receptors.

### vFLIP is readily degraded by autophagy in MAVS-deficient BCBL-1 cells

**Fig 2:**
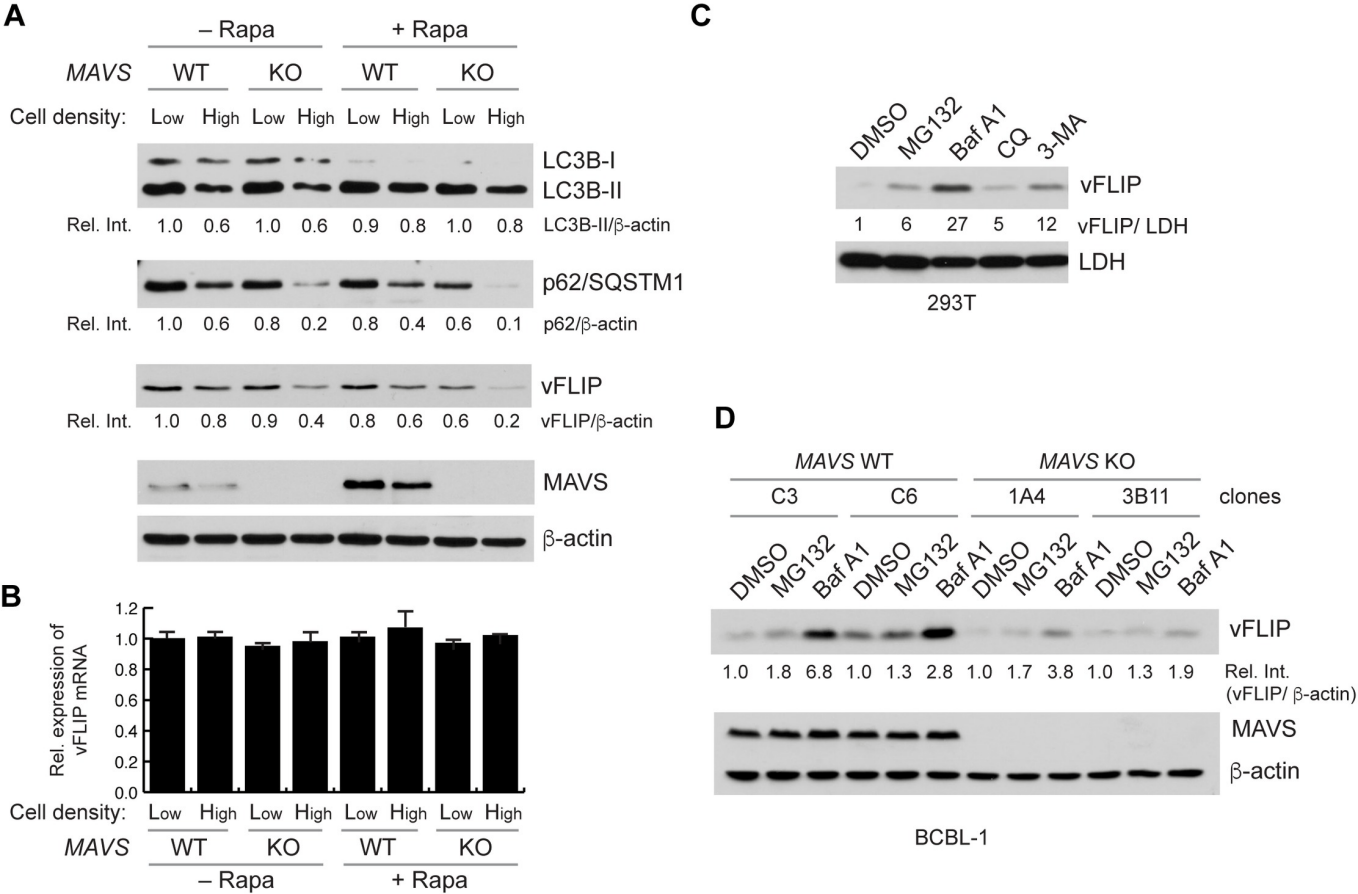
Autophagy-induced vFLIP degradation. (A) Immunoblots of LC3B, p62, vFLIP, MAVS, and β-actin from WT and *MAVS* KO BCBL-1 cells cultured at low (seeded at 5 x 10^4^ cells/ml) and high (seeded at 2 x 10^5^ cells/ml) densities in the presence and absence of 20 nM rapamycin for 2 days. Relative band intensities normalized by β-actin were calculated and noted below each panel. (B) Real-time quantitative PCR (RT-qPCR) analysis of vFLIP mRNA from the cells cultured as in (A). Data are represented as mean ± SD of triplicate. (C) Immunoblots of vFLIP and loading control lactate dehydrogenase (LDH) from vFLIP-transfected 293T treated with 10 μM MG132, 100 nM bafilomycin A1 (Baf A1), 5 μM chloroquine (CQ), and 5 mM 3-methyladenine (3-MA) for 1 day. Relative vFLIP intensities normalized by LDH are shown below each panel. (D) Immunoblots of vFLIP, MAVS, and loading control β-actin from WT and *MAVS* KO BCBL-1 cell lines cultured at high density for 2 days and then treated with 20 nM Baf A1 and 10 μM MG-132 for 8 h. Relative vFLIP intensities normalized by β-actin are shown below each panel.

Upon autophagy induction, LC3-I, a cytosolic form of LC3 (one of mammalian ATG8 orthologs), is cleaved, lipidated, and inserted as LC3-II into autophagosome membranes, and the selective autophagy receptor p62 (also termed SQSTM1) is degraded. Thus, autophagy can be assessed by immunoblotting for the faster migrating LC3-II and p62 levels [29]. Intriguingly, our data showed that high-density culture led to a decrease in LC3B-II level in the WT and *MAVS* KO cells (Fig 2A). This might reflect an increase in autophagic flux and lysosomal degradation of LC3B-II in a MAVS-independent manner. Rapamycin promoted a pronounced increase in the relative levels of LC3B-II under both low- and high-density cultures, but there was no difference between the isogenic cell lines (Fig 2A). Nonetheless, p62 degradation was highly induced by high-density culture and/or rapamycin in *MAVS* KO BCBL-1 cells (Fig 2A). By contrast, this did not occur in *MAVS* KO BJAB and EBV-infected AKATA cells (S3 Fig). These results suggest that MAVS may be involved in the inhibition of autophagy and associated cell death induced by HHV-8 infection.

HHV-8 vFLIP was proposed to function as a key regulator of autophagy and associated cell death [32]; therefore, we examined the expression level of vFLIP. The result showed that the level of vFLIP protein, but not mRNA, was reduced by high-density culture and/or rapamycin in *MAVS* KO BCBL-1 cells in an analogous manner to p62 (Fig 2A and 2B). Thus, autophagy and cell death induced in *MAVS* KO BCBL-1 cells may be related to reduced levels of vFLIP. Levels of vFLIP were increased in the presence of autophagy inhibitors, in particular bafilomycin A1 (Baf A1), as well as a proteasome inhibitor MG132, to a lesser extent, in vFLIP-transfected 293T and BCBL-1 cells under high-density culture (Fig 2C and 2D, respectively). Importantly, the inhibitors restored vFLIP expression in *MAVS* KO BCBL-1 cells (Fig 2D), indicating that MAVS deficiency-induced vFLIP degradation is mediated by autophagy and the ubiquitin-proteasome pathway. Of note, the effect of MG132 was relatively weaker than that of Baf A1, indicating that autophagy is the main mechanism of vFLIP degradation with a minor contribution from the proteasome pathway. These results suggest that MAVS may be involved in the protection of vFLIP from degradation mediated by autophagy and proteasomes.

### MAVS selectively stabilizes vFLIP

**Fig 3:**
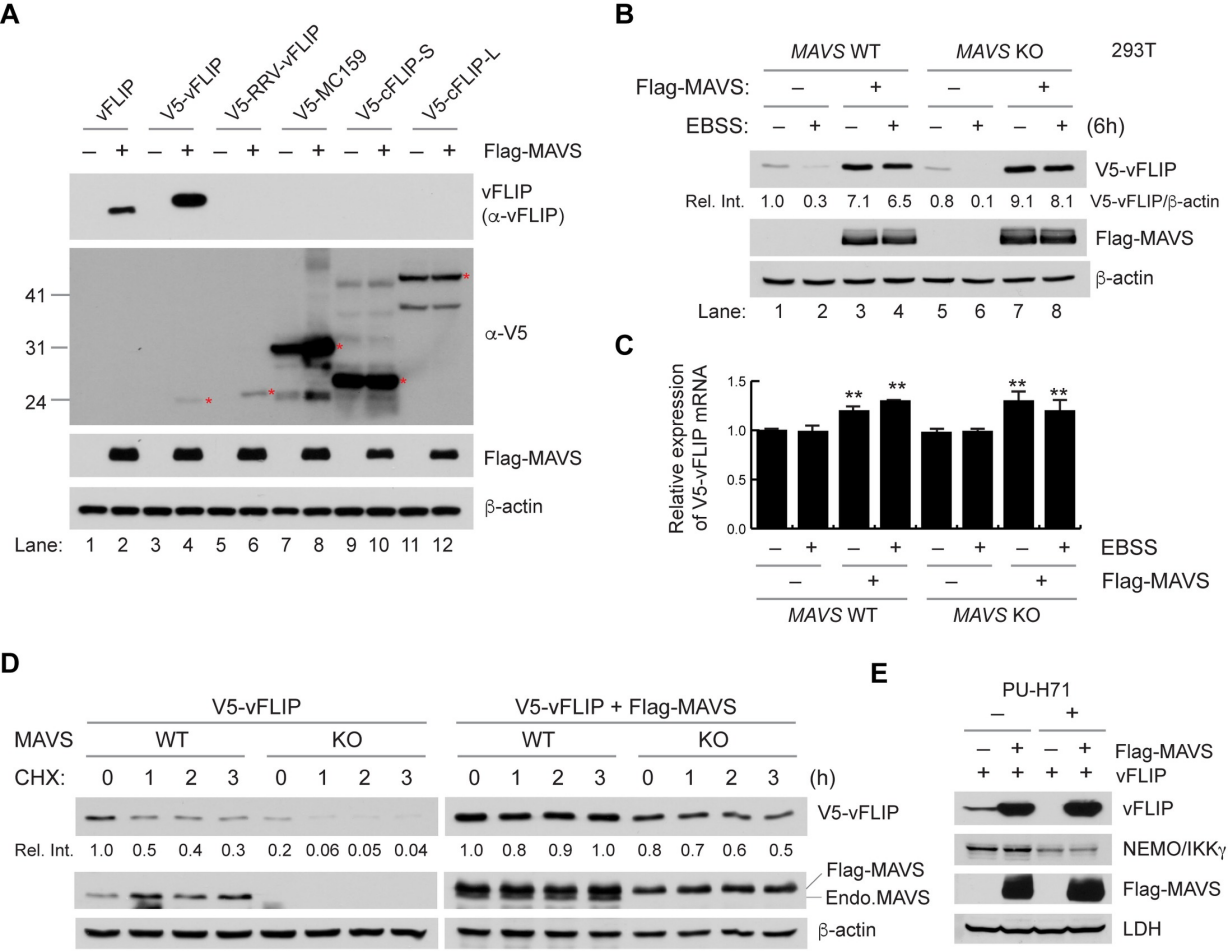
MAVS selectively stabilizes vFLIP. (A) Immunoblots of extracts of 293T cells transfected with non- or V5-tagged viral and cellular FLIPs along with empty or Flag-MAVS vectors for 24 h. Red asterisks indicate bands of expected molecular weight. (B) Immunoblots of extracts from WT and *MAVS* KO 293T cells transfected with V5-vFLIP and either empty vector or Flag-MAVS and incubated in EBSS and complete media (–EBSS) for 6 h. Relative V5-vFLIP intensities normalized to β-actin were noted under each band. (C) Real time-quantitative PCR analysis of V5-vFLIP mRNA expression. Total RNAs were isolated from the cells transfected and treated as in (B) above. Data are represented as mean ± SD of triplicate samples. (**p<0.05) (D) Immunoblots of extracts from WT and *MAVS* KO 293T cells transfected with V5-vFLIP and either empty vector or Flag-MAVS and treated with cycloheximide (CHX) for 0, 1, 2, and 3 h. “Endo.MAVS” indicates endogenous MAVS. (E) Immunoblots of extracts from 293T cells transfected with vFLIP and either empty vector or Flag-MAVS and treated with DMSO or 1 μM PU-H71 for 24 h.

We next examined if overexpression of MAVS has an influence on vFLIP expression in 293T cells. The results indicated that vFLIP, expressed alone, was barely detected, but its level was significantly increased when MAVS was co-expressed (Fig 3A, compare lanes 1 and 2). To facilitate the specific detection of vFLIP in co-immunoprecipitation (co-IP) and immunostaining assays, we generated vFLIP vectors for expression of vFLIP fused to various N-terminally placed epitope tags. Expression of the vFLIP proteins was tested in the absence and presence of MAVS. Surprisingly, basal expression levels of the tagged vFLIP proteins, except for V5-tagged vFLIP (V5-vFLIP) (Fig 3A, lanes 3 and 4), were elevated relative to native vFLIP and not significantly increased by MAVS co-expression (S4 Fig). Therefore, we used V5-vFLIP for subsequent experiments. We next examined the effect of MAVS on expression of other viral and cellular FLIPs including rhesus monkey rhadinovirus (RRV) vFLIP, poxvirus molluscum contagiosum MC159, and cellular FLIP long form (cFLIP-L) and short form (cFLIP-S). Intriguingly, MAVS increased the expression of the viral FLIPs but not the cellular FLIPs (Fig 3A, lanes 5 to 12), suggesting that MAVS selectively regulates expression of viral FLIP proteins. We next examined whether MAVS overexpression can inhibit autophagy-induced vFLIP degradation. As expected, EBSS-induced autophagy highly promoted V5-vFLIP degradation in WT and *MAVS* KO 293T cells [26] (Fig 3B, compare lanes 1 and 5 with lanes 2 and 6, respectively). However, the EBSS-induced vFLIP degradation was significantly blocked by MAVS overexpression in WT and *MAVS* KO cells (Fig 3B, compare lanes 3 and 7 with lanes 4 and 8, respectively). While the mRNA levels of transfected V5-vFLIP were comparable between the isogenic cell lines, MAVS overexpression slightly increased the mRNA levels to ~1.2 fold (Fig 3C). Thus, we performed a cycloheximide (CHX) chase experiment to verify that MAVS increases the expression level of V5-vFLIP protein by stabilization rather than by enhanced mRNA translation or abundance. The result showed that V5-vFLIP protein was highly unstable in MAVS-deficient cells but stabilized by endogenous MAVS and even more so by transfected MAVS (Fig 3D). Using the HSP90 inhibitor PU-H71, it was recently demonstrated that this heat-shock protein plays a crucial role in stabilization of vFLIP [33]. PU-H71-induced vFLIP degradation indeed occurred in 293T cells but was inhibited by MAVS (Fig 3E). However, MAVS could not block PU-H71-induced degradation of NEMO/IKKγ, another client of HSP90 (Fig 3E). Collectively, these results suggest that MAVS selectively stabilizes vFLIP.

### MAVS stabilizes vFLIP via TRAF proteins

**Fig 4:**
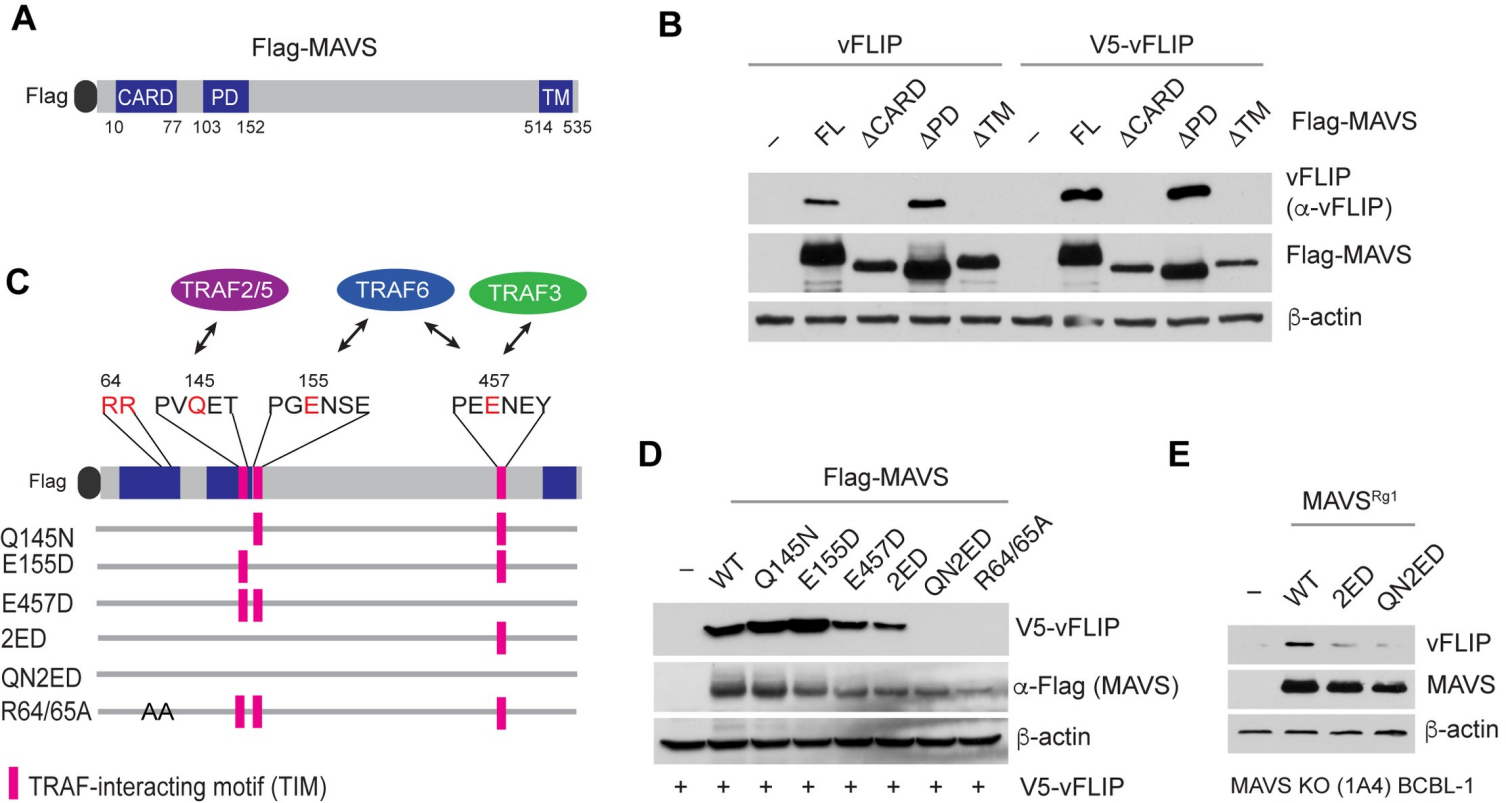
MAVS stabilizes vFLIP via the CARD region and TRAF-interacting motifs. (A) Diagram of Flag-MAVS showing the position of its caspase activation and recruitment domain (CARD), proline-rich domain (PD), and transmembrane (TM) regions. (B) Immunoblots of extracts from 293T cells transfected with Flag-MAVS full-length (FL) and deletion mutants (ΔCARD, ΔPD, and ΔTM) together with non-tag vFLIP or V5-vFLIP. (C) Diagram of MAVS mutations of the CARD and TRAF-interacting motifs (TIMs). (D) Immunoblots of extracts from 293T cells transfected with Flag-MAVS WT and point mutants together with V5-vFLIP. (E) Immunoblots of extracts from BCBL-1 *MAVS* KO (1A4) cells lentivirally transduced with WT and mutants (2ED and QN2ED) of MAVS (MAVS^Rg1^) resistant to the MAVS gRNA1.

To identify the region(s) of MAVS required for vFLIP stabilization, three truncated forms of MAVS lacking the caspase activation and recruitment domain (CARD)-like domain (residues 10 to 77, ΔCARD), the proline-rich domain (residues 103 to 152, ΔPD), or the transmembrane domain (residues 514 to 535, ΔTM) were used (Fig 4A) as previously described [26]. Expression of both non-tagged vFLIP and V5-vFLIP were increased by full-length (FL) and ΔPD MAVS, but not by ΔCARD and ΔTM MAVS (Fig 4B), indicating that vFLIP stabilization requires the CARD and TM regions.

Since the CARD and TM regions are essential for MAVS downstream signaling [34] and TNF receptor associated factor 6 (TRAF6) plays a role in regulation of protein stability [35], we reasoned that the MAVS-TRAF signaling complex may be involved in vFLIP stabilization. MAVS contains multiple TRAF-interacting motifs (TIMs) for recruiting TRAF2, TRAF3, TRAF5, and TRAF6 [12, 36] (Fig 4C): the PVQET (residues 143–147) motif for TRAF2 and TRAF5, the PGENSE (residues 153–158) and PEENEY (residues 455–460) motifs for TRAF6, and the second TRAF6-binding motif also for TRAF3. MAVS mutations that selectively disrupt its binding to specific TRAFs were generated and used for vFLIP stabilization assays. Single or double mutations of TRAF2/5 (Q145N), TRAF6 (E155D, E457D, or both (2ED)), TRAF3/6 (E457D) binding sites had no or little effect on vFLIP stabilization, but mutations of all the TIMs (QN2ED) abolished the ability of MAVS to stabilize vFLIP (Fig 4D). The CARD region is essential for MAVS polymerization, which is required for recruitment of TRAFs [12]. A polymerization-null CARD mutation (R64/65A) abolished the ability of MAVS to stabilize vFLIP (Fig 4D). In addition, reconstitution of WT but not the mutants (2ED and QN2ED) of MAVS^Rg1^, which is a codon-degenerated version of MAVS that is resistant to the guide RNA1, into BCBL-1 *MAVS* KO 1A4 led to stabilization of endogenous vFLIP (Fig 4E). These results suggest that TRAFs, in a redundant manner, are critically involved in MAVS-induced vFLIP stabilization.

### MAVS mediates TRAF-induced vFLIP stabilization

**Fig 5:**
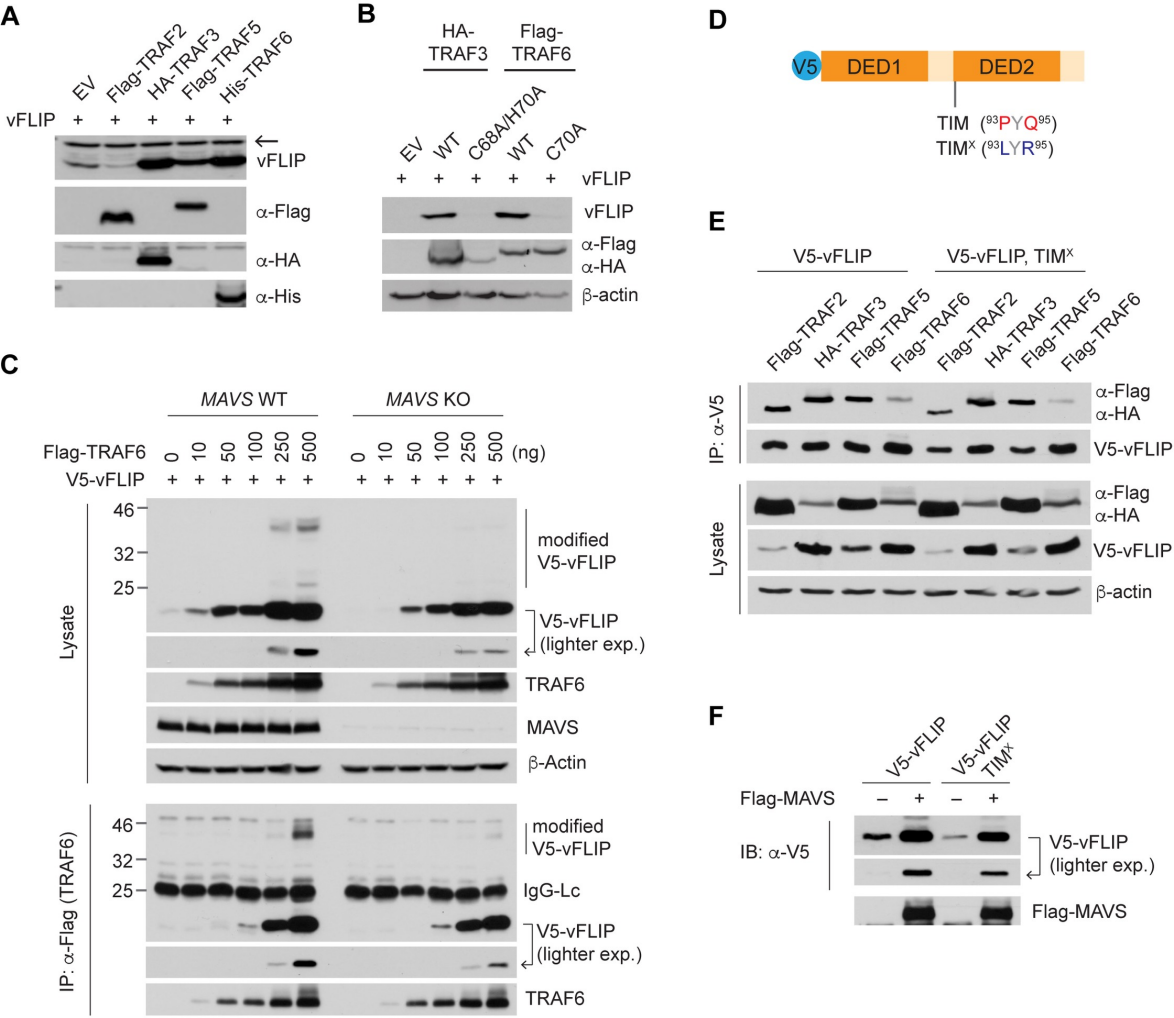
MAVS is involved in TRAF-induced vFLIP stabilization. (A-B) Immunoblots of extracts from 293T cells transfected with the indicated TRAFs and vFLIP (A) and with WT and catalytic mutant of HA-TRAF3 or Flag-TRAF6 along with vFLIP (B). Arrow indicates a band of unknown identity, which was used as a loading control. (C) Immunoblotting and co-immunoprecipitation (co-IP) analyses of extracts from WT and *MAVS* KO 293T cells transfected with 500 ng V5-vFLIP and the indicated amounts of Flag-TRAF6 vector. IgG-Lc indicates light chain of immunoglobulin. (D) Diagram of V5-vFLIP showing the position of a TRAF-interacting motif (TIM) and its mutation (TIM^X^), in which P93 and Q95 were substituted with leucine (L) and arginine (R), respectively. (E) Co-IP assay with extracts from 293T cells transfected with V5-vFLIP WT and TIM^X^ mutant along with the indicated TRAF plasmids. (F) Immunoblots of extracts from 293T cells transfected with V5-vFLIP WT and TIM^X^ mutant in the presence and absence of Flag-MAVS.

We next examined the effect of each individual TRAF on vFLIP expression in 293T cells. The results showed that TRAF3, TRAF5, and TRAF6, but not TRAF2, increased vFLIP expression (Fig 5A). TRAF proteins are ubiquitin ligases containing a highly conserved N-terminal RING domain forming the catalytic site; TRAF1 is the exception in that it does not contain this conserved domain. To further examine whether the catalytic activity of the TRAF proteins is required for vFLIP stabilization, we used TRAF3 (C68A/H70A) and TRAF6 (C70A) RING domain mutants that lack E3 ligase activity [37, 38]. Indeed, the TRAF mutants completely lost the ability to enhance vFLIP expression (Fig 5B), suggesting that vFLIP may be posttranslationally modified by TRAF-mediated K63-linked polyubiquitination on a specific lysine residue(s) for stabilization. To next examine if MAVS mediates TRAF-induced vFLIP expression, WT and *MAVS* KO 293T cells were co-transfected with V5-vFLIP and TRAF6 expression vectors. TRAF6 promoted the modification of V5-vFLIP as observed by the slower migrating bands, possibly K63-linked polyubiquitinated products, but this effect was significantly diminished in the *MAVS* KO cells (Fig 5C). Transfection efficiency was comparable between the isogenic cell lines as evidenced by the expression of Flag-TRAF6. In addition, RT-qPCR analysis showed that there was no difference in the mRNA levels of V5-vFLIP between WT and *MAVS* KO cells co-transfected with TRAF6, while TRAF6 overexpression modestly increased the mRNA levels of V5-vFLIP (S5 Fig). This increase in the level of V5-vFLIP mRNA may contribute to induction of V5-vFLIP protein in the *MAVS* KO cells despite the apparent absence of post-translational modifications. Taken together, these results indicate that MAVS promotes TRAF-induced post-translational modification of vFLIP for stabilization. To better understand the molecular mechanism by which MAVS promotes TRAF6-induced vFLIP stabilization, we examined if MAVS mediates an interaction of vFLIP and TRAF6. Indeed, our co-IP assay showed that V5-vFLIP was co-precipitated with Flag-TRAF6 in the presence of MAVS and the interaction was diminished in *MAVS* KO cells (Fig 5C). Importantly, TRAF6 interaction with the modified V5-vFLIP proteins was readily apparent in control cells but not in *MAVS* KO cells (Fig 5C), suggesting that MAVS mediates TRAF6-induced polyubiquitination of vFLIP.

It has been reported that vFLIP contains a TRAF-interacting motif (TIM) at the N-terminal region of the death effector domain 2 (DED2) (Fig 5D) and binds preferentially to TRAF2 via the motif [39]. Thus, it is conceivable that direct binding of TRAF proteins also contributes to vFLIP stabilization and modification. To examine this possibility, we generated a variant of vFLIP (TIM^X^) in which the consensus residues, proline 93 (P93) and glutamine 95 (Q95), of the TIM were substituted with leucine (L) and arginine (R), respectively (Fig 5D). Then, we examined if vFLIP TIM^X^ indeed loses the ability to bind to TRAF proteins using a co-IP assay. Contrary to our expectation, however, the result showed that the TIM^X^ variant could bind to TRAF3 and TRAF5, and to a lesser extent to TRAF2 and TRAF6 (Fig 5E). In addition, MAVS-induced vFLIP stabilization was not significantly affected by the mutation (Fig 5F). These results suggest that vFLIP contains other unknown TIM motif(s) and/or binds indirectly to the TRAF proteins via an adaptor such as MAVS (Fig 5C). Taken together, our results suggest that TRAFs promote vFLIP stabilization via MAVS rather than by direct binding to vFLIP. However, the precise molecular mechanism by which MAVS utilizes TRAF proteins for vFLIP stabilization remains to be determined.

**Fig 6:**
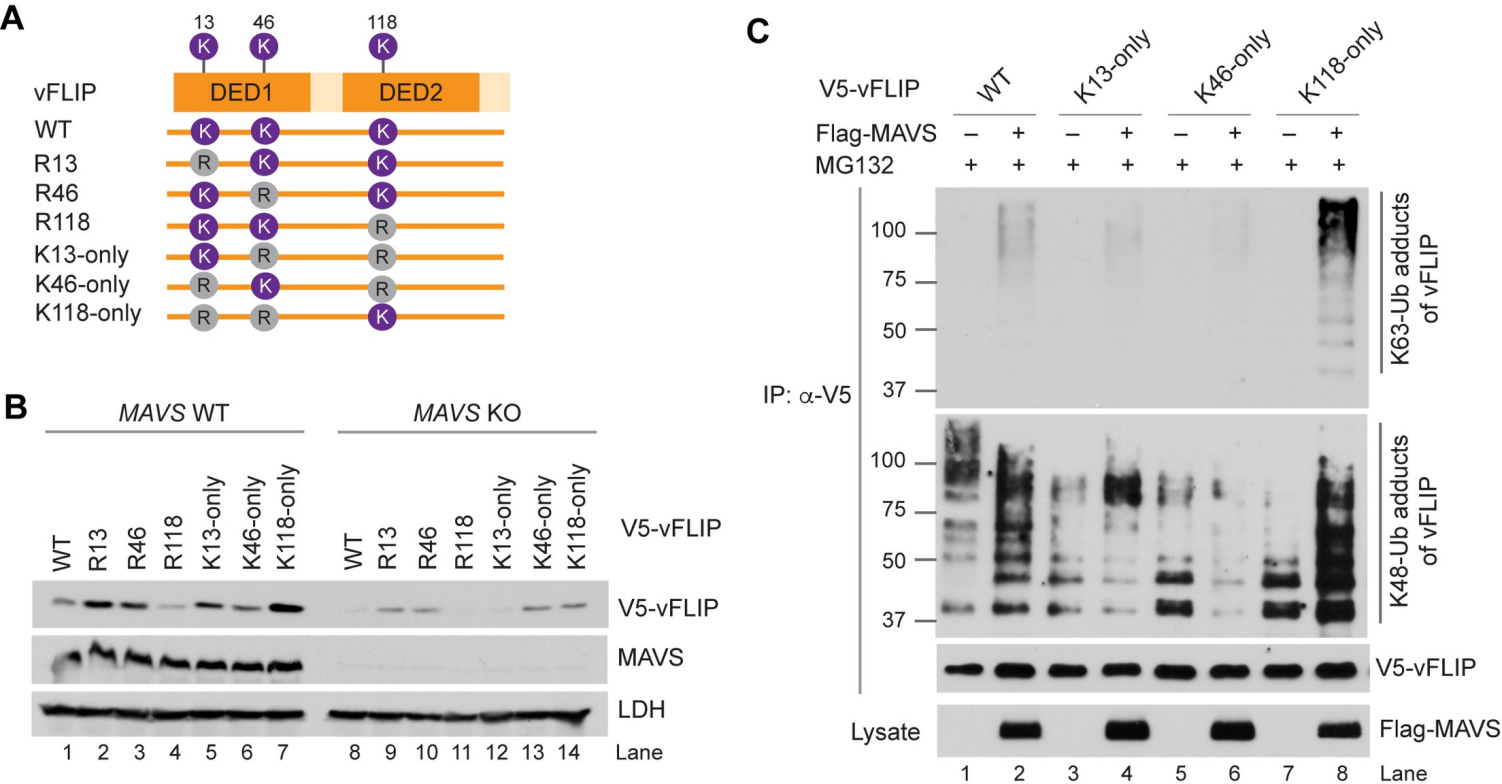
MAVS promotes K63-linked polyubiquitination at lysine 118 of vFLIP. (A) Diagram of the single and combined vFLIP mutations in lysine residues at positions 13, 46, and 118. (B) Immunoblots of extracts from WT and *MAVS* KO 293T cells transfected for 24 h with expression vectors for WT V5-vFLIP and the indicated mutants of V5-vFLIP. (C) *In vivo* vFLIP ubiquitination assays using 293T cells transfected with vectors expressing WT or lysine-mutated V5-vFLIP with or without Flag-MAVS in the presence of 10 μM MG-132 for 24 h.

vFLIP contains three lysine residues at positions 13, 46, and 118. To test the involvement of these lysines in MAVS-mediated vFLIP stabilization, we generated single or double lysine mutants of vFLIP by substituting lysine (K) for arginine (R) (Fig 6A): R13 (K13R), R46 (K46R), R118 (K118R), K13-only (K46R/K118R), K46-only (K13R/K118R), and K118-only (K13R/K46R). We first compared the expression levels of vFLIP WT and lysine mutants in control and *MAVS* KO 293T cells. Basal expression of vFLIP was significantly reduced in the *MAVS* KO cells (Fig 6B, compare lanes 1 and 8). K118-only vFLIP exhibited the highest expression (Fig 6B, lane 7), but R118 vFLIP showed the lowest expression in control 293T cells (Fig 6B, lane 4), suggesting that K118 may be centrally involved in vFLIP stabilization. Nonetheless, K118-only vFLIP was still readily detected in the MAVS-deficient cells (Fig 6B; compare lanes 8 and 14), suggesting the possibility that either (1) there is an alternative MAVS-independent pathway for vFLIP stabilization via K118; or (2) the other lysines, K13 and K46, mediate proteasomal degradation of vFLIP via K48-linked polyubiquitination, thus their mutation may result in stabilization of K118-only vFLIP independently of MAVS. Indeed, R13 and R46 vFLIP proteins were more highly expressed compared to vFLIP in the absence or presence of MAVS (Fig 6B; compare lanes 2 and 3 with lane 1 and lanes 9 and 10 with lane 8). We next performed ubiquitination assays to examine how MAVS modulates K48- and K63-linked polyubiquitination of vFLIP. MG132 was included in the assays to prevent vFLIP degradation. In line with our hypothesis, MAVS could strongly induce K63-linked polyubiquitination of vFLIP at K118, but not at K13 and K46 residues (Fig 6C). Compared to K118-only vFLIP, vFLIP exhibited less K63-linked polyubiquitination, implying that K13 and K46 might be involved in the repression of K63-linked polyubiquitination at K118. Intriguingly, K48-linked polyubiquitinated vFLIP bands above ~150 kDa disappeared when MAVS was overexpressed; instead, the area was replaced by K63-linked polyubiquitinated vFLIP proteins (Fig 6C, compare lanes 1 and 2). Overall, these results suggest that MAVS can stabilize vFLIP by promoting K63-linked polyubiquitination, mainly at K118.

### vFLIP binds directly to and is stabilized by peroxisome-localized MAVS

**Fig 7:**
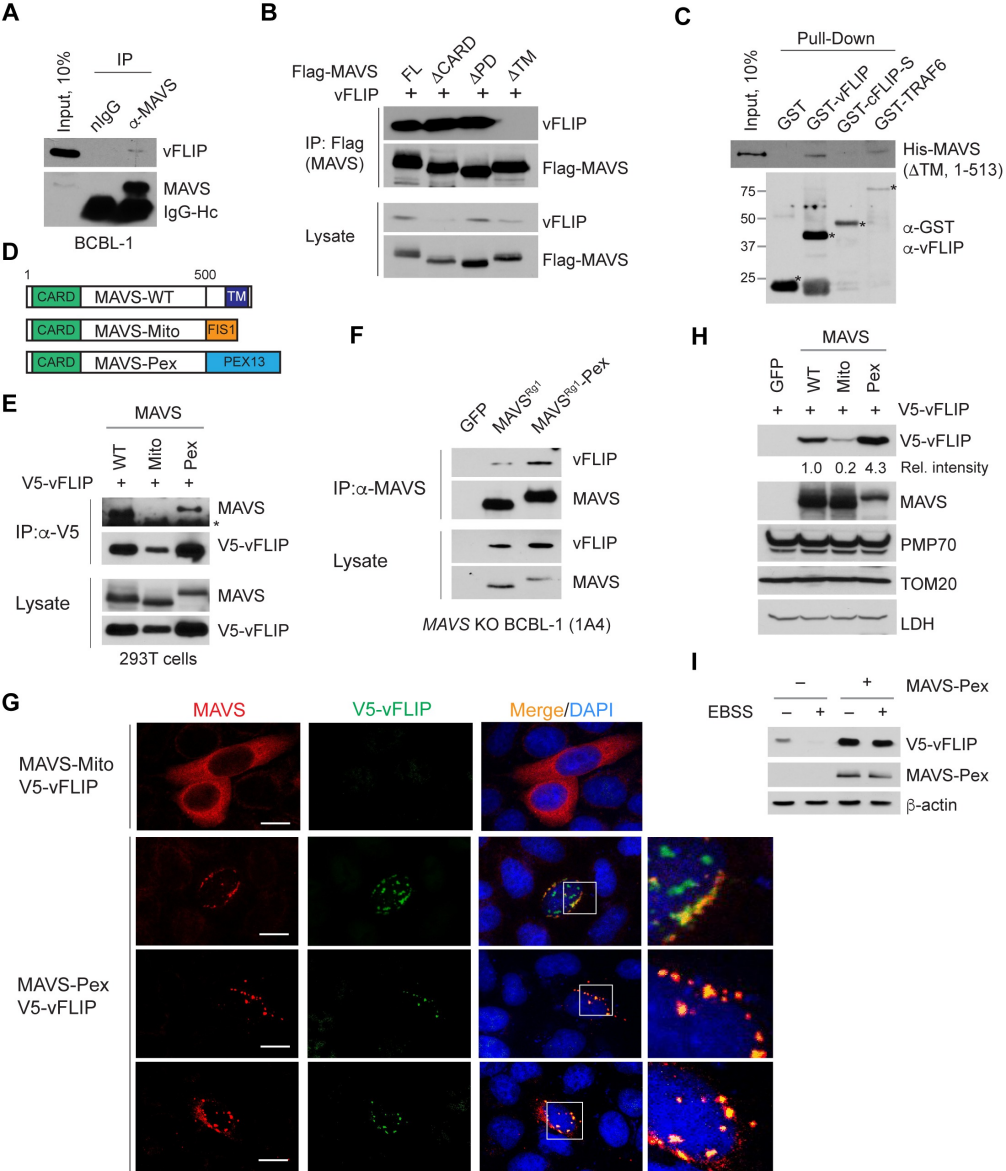
vFLIP binds to and is stabilized by peroxisome-localized MAVS. (A-B) Co-IP assays using extracts from BCBL-1 cells (A) and 293T cells transfected with vectors expressing Flag-MAVS or deletion mutants together with vFLIP (B). IgG-Hc indicates heavy chain of immunoglobulin. (C) *In vitro* GST pull-down assay using the indicated purified recombinant proteins. Since GST-vFLIP was poorly detected by anti-GST antibody, the same membrane was re-probed with anti-vFLIP antibody. Asterisks indicate intact GST-fusion proteins. (D) Diagram of MAVS, MAVS-Mito, and MAVS-Pex constructs. (E-F) Co-IP assays using extracts from *MAVS* KO 293T cells transfected with the indicated MAVS and V5-vFLIP vectors (E) and of *MAVS* KO BCBL-1 (1A4) reconstituted with GFP, MAVS^Rg1^, and MAVS^Rg1^-Pex (F). Asterisk indicates heavy chain of immunoglobulin. (G) Immunostaining of *MAVS* KO 293T cells transfected with the indicated MAVS and V5-vFLIP vectors. Co-localization (yellow spots) of MAVS-Pex and V5-vFLIP is shown in the enlarged inset. (H) Immunoblots of extracts from *MAVS* KO 293T cells transfected with V5-vFLIP and the indicated MAVS or GFP vectors. Relative vFLIP band intensities normalized by LDH are marked below the blot. (I) Immunoblots of extracts from *MAVS* KO 293T cells transfected with V5-vFLIP either with or without MAVS-Pex and incubated in complete media and EBSS for an additional 6 h.

Selective regulation of vFLIP stability by MAVS is likely to be mediated by their physical interaction. We first performed a co-IP assay to examine intracellular interaction between endogenous vFLIP and MAVS proteins in BCBL-1 cells. Indeed, vFLIP was detected in the MAVS-IP complex but not in normal immunoglobulin (nIgG)-IP (Fig 7A). To next identify the region of MAVS required for vFLIP binding, we co-transfected 293T with full-length MAVS and the three deletion variants used in Fig 4B together with vFLIP. Co-IP analysis showed that vFLIP interacted with full-length, ΔCARD, ΔPD MAVS, but not with ΔTM MAVS (Fig 7B). Thus, the interaction with vFLIP specifically required the TM domain, but not the CARD and PD regions, of MAVS. However, a pull-down assay revealed that purified GST-vFLIP, but not GST or GST-cFLIP-S, still bound to the purified recombinant MAVS (residues 1–513) lacking the TM domain (Fig 7C). GST-TRAF6 was used as a positive control for MAVS binding. These results suggest that vFLIP may bind directly to a region other than the CARD, PD, and TM regions of a membrane-bound form of MAVS.

MAVS is known to localize on the surfaces of mitochondria and peroxisomes [16, 18]; ~80% of MAVS localizes to mitochondria and 3–20% to peroxisomes. On the other hand, mitochondrial or peroxisomal localization of vFLIP has not been reported. To test if vFLIP binds to mitochondrial or peroxisomal MAVS, we performed a co-IP assay using *MAVS* KO 293T cells co-transfected with V5-vFLIP and MAVS, mitochondria-targeting MAVS (MAVS-Mito), or peroxisome-targeting MAVS (MAVS-Pex) (Fig 7D), which were described in a previous study [16]. Interestingly, MAVS-Pex as well as MAVS were readily detected in the V5-vFLIP-IP complex, but MAVS-Mito was barely detected (Fig 7E). Although the failure to detect a strong interaction of vFLIP with MAVS-Mito may be due in part to a lower expression of vFLIP (Fig 7E), our results raise the intriguing possibility that vFLIP interacts with and is stabilized preferentially by peroxisome-localized MAVS. The low level of endogenous vFLIP detected in the co-IP assay of Fig 7A may be due to the relatively low amount of peroxisomal MAVS. To further test this possibility, we reconstituted *MAVS* KO BCBL-1 cells with MAVS^Rg1^-Pex and performed a co-IP experiment. As expected, the result indicated that more vFLIP was detected in MAVS^Rg1^-Pex-IP than MAVS^Rg1^-IP (Fig 7F). Furthermore, an immunostaining assay showed that vFLIP was readily detected in MAVS-Pex-reconstituted cells and co-localized with MAVS-Pex, but not with MAVS-Mito in reconstituted cells (Fig 7G). In line with these findings, quantitative immunoblotting analysis indicated that MAVS-Pex increased V5-vFLIP expression by more than 4-fold compared to MAVS, whereas MAVS-Mito diminished V5-vFLIP expression to 20% of WT MAVS levels (Fig 7H). As loading controls, peroxisomal and mitochondrial markers PMP70 and TOM20 exhibited consistent expression. Furthermore, MAVS-Pex could protect vFLIP from autophagy-induced degradation (Fig 7I). Since TRAF6 is implicated in MAVS-induced vFLIP stabilization (Fig 5), we examined if TRAF6 can localize to peroxisomes using immunostaining. The results showed that TRAF6 localized in part to peroxisomes in control 293T cells, and less so in *MAVS* KO cells (S6 Fig). However, when co-transfected with MAVS-Pex, TRAF6 was highly detected in peroxisomes together with MAVS-Pex (S6 Fig), indicating that peroxisomal targeting of TRAF6 may be dependent on peroxisome-localized MAVS.

### vFLIP is targeted to and is active on peroxisomes

**Fig 8:**
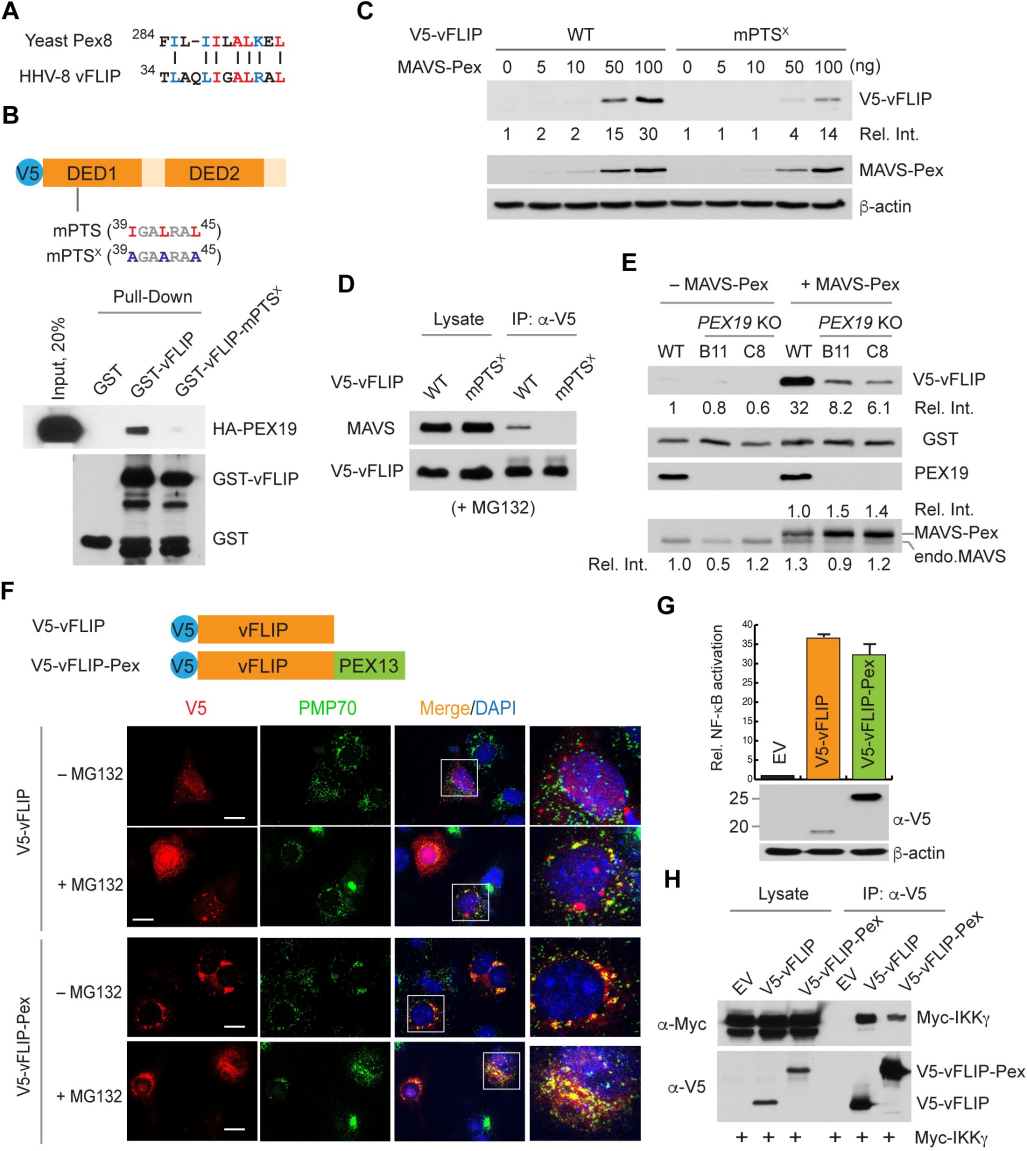
Functional vFLIP is targeted to peroxisomes in a PEX19-dependent manner. (A) Alignment of putative mPTSs of yeast Pex8 and vFLIP (residues 34–45). Identical and similar residues are highlighted in red and blue, respectively. (B) *In vitro* GST pull-down assay using purified GST, GST-vFLIP, and GST-vFLIP mPTS^X^ proteins and the extracts from 293T cells expressing HA-PEX19. (C) Immunoblots of extracts from *MAVS* KO 293T cells transfected with V5-vFLIP WT and mPTS^X^ along with the indicated amounts of MAVS-Pex vectors. Relative V5-vFLIP band intensities normalized by β-actin are shown below the blot. (D) Co-IP assay using extracts from 293T cells transfected with V5-vFLIP WT and mPTS^X^ and treated with 10 μM MG132 for 8 h. (E) Immunoblots of extracts from WT and *PEX19* KO 293A cell lines transfected with vectors expressing V5-vFLIP and GST with or without MAVS-Pex vectors. Relative V5-vFLIP band intensities normalized by GST are shown below the blot. “Endo.MAVS” indicates endogenous MAVS. Relative MAVS-Pex and endogenous MAVS band intensities are shown above and below the MAVS immunoblot panel, respectively. (F) Immunostaining of 293A cells transfected with V5-vFLIP and V5-vFLIP-Pex. DMSO or MG132 was added at 8 h to the cell culture before fixation. The insets show enlarged portions of the images, revealing co-localization or juxtaposition of V5-vFLIP with peroxisomal marker PMP70. (G) NF-κB reporter assay in 293T cells transfected with empty vector, V5-vFLIP, and V5-vFLIP-Pex vectors together with an NF-κB-luciferase reporter plasmid. The corresponding cell extracts were then analyzed by immunoblotting with anti-V5 and β-actin antibodies. Data are presented as mean ± SD of triplicate samples. (H) Co-IP assay using extracts from 293T cells transfected with Myc-IKKγ expression plasmid and empty vector, V5-vFLIP, or V5-vFLIP-Pex vectors.

Peroxisome-specific stabilization of vFLIP by MAVS might be explained by selective targeting of vFLIP to peroxisomes. Our inspection of the vFLIP primary structure using the Blocks-Based Tools [40] revealed a region homologous to a putative PEX19 binding site (PEX19BS) of yeast Pex8 (Fig 8A). PEX19 is a peroxisomal biogenesis factor that delivers cargo proteins to the peroxisomal membrane by binding to the PEX19BS motif, also termed the membrane peroxisomal targeting signal (mPTS) sequence, of cargo [41]. To determine whether the predicted vFLIP mPTS motif can bind to PEX19, we performed a pull-down assay using GST-fused WT and mutant (mPTS^X^) vFLIPs, the latter containing alanine substitutions of conserved hydrophobic residues I39, L42, and L45 (Fig 8B). Indeed, GST-vFLIP, but not GST or the vFLIP mutant, bound to HA-PEX19 derived from transfected 293T cells (Fig 8B). Next, we examined the effect of MAVS on the expression of vFLIP mPTS^X^. *MAVS* KO 293T cells were transfected with V5-vFLIP WT and mPTS^X^ along with different amounts of MAVS-Pex vector. The result showed that MAVS-Pex stabilization of mPTS^X^ was diminished relative to its effect on vFLIP (Fig 8C). Consistent with this result, vFLIP mPTS^X^ binding to endogenous MAVS was significantly diminished (Fig 8D). To further examine the effect of PEX19 on vFLIP expression, we generated two *PEX19* KO 293A cell lines, B11 and C8, using CRISPR/Cas9-mediated gene editing. Indeed, basal and MAVS-Pex-induced expression of vFLIP were significantly reduced in the PEX19-deficient cells even though MAVS-Pex expression was increased up to 50% in PEX19-deficient cells compared to its expression in the WT cells (Fig 8E). The expression of endogenous MAVS was not significantly affected by PEX19 deficiency. The transfection efficiency was comparable among the isogenic cell lines as evidenced by the expression of co-transfected GST (Fig 8E). Intriguingly, MAVS-Pex still marginally increased the expression of V5-vFLIP in *PEX19* KO cells (Fig 8E). This might be attributed to incomplete knockout of peroxisomes in *PEX19* KO cells. Of note, our immunostaining results revealed that PMP70, a cargo molecule of PEX19, was largely, but not completely, depleted in *PEX*19 KO cells (S7 Fig). Consistent with this, V5-vFLIP WT and mPTS^X^ were not readily detected in *PEX19* KO cells, even in the presence of MAVS-Pex; however, V5-vFLIP, but not mPTS^X^, was readily detected in peroxisomes of control cells when co-transfected with MAVS-Pex (S7 Fig). Taken together, these data indicate, for the first time, that vFLIP can be targeted to peroxisomes where it is stabilized by MAVS.

One of the best characterized functions of vFLIP is its activation of NF-κB. To determine if peroxisome-localized vFLIP can activate NF-κB, we generated a chimeric vFLIP-Pex construct in which the human PEX13 PTS sequences (residues 145–233) were fused to the C-terminus of vFLIP (Fig 8F). We first examined peroxisomal localization of the vFLIP protein in 293A cells by immunostaining; V5-vFLIP-Pex was strongly co-localized with PMP70 while co-localization or juxtaposition of V5-vFLIP and PMP70 was readily detected only in the presence of MG132 (Fig 8F). Using a luciferase-based reporter assay for NF-κB signaling, we determined that V5-vFLIP-Pex can activate NF-κB in 293T cells (Fig 8G). However, this result does not necessarily indicate that peroxisomes are a unique site for vFLIP activation of NF-κB [42]; indeed, equivalent levels of NF-κB activation by V5-vFLIP and V5-vFLIP-Pex, despite higher expression of the latter, may indicate that this is not the case (Fig 8G). To further demonstrate the ability of peroxisomal vFLIP to activate NF-κB, we examined if vFLIP-Pex interacts with NEMO/IKKγ, which is essential for vFLIP-induced NF-κB activation [43, 44], using a co-IP assay. Indeed, vFLIP-Pex interacted with NEMO/IKKγ, but to a lesser extent than vFLIP (Fig 8H). Thus, our results demonstrate a novel and essential role of peroxisomes as platforms for vFLIP stabilization and that peroxisome-localized vFLIP can activate NF-κB signaling.

### Targeted disruption of vFLIP-MAVS interaction promotes the death of HHV-8-infected cells

**Fig 9:**
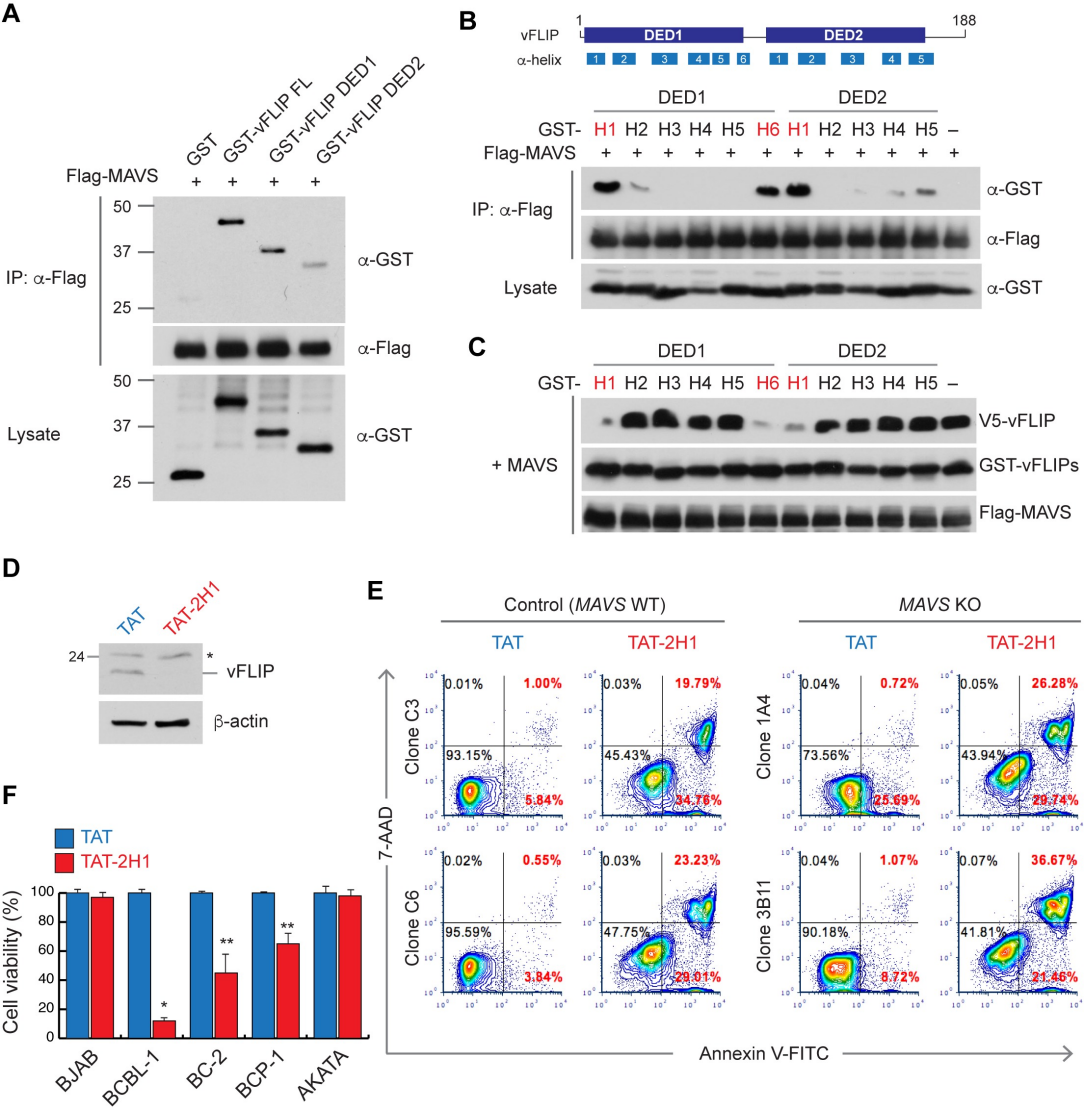
Functional interaction of vFLIP and MAVS is essential for survival of HHV-8-infected PEL cells. (A-B) Co-IP assay using extracts from 293T cells transfected with expression vectors for Flag-MAVS and (A) GST, GST-vFLIP, GST-DED1, or GST-DED2 and (B) GST and GST-vFLIP helices. (C) Immunoblots of extracts from 293T cells transfected with V5-vFLIP and Flag-MAVS together with GST-vFLIP helices. (D) Immunoblots of extracts from BCBL-1 cells treated with 10 μM TAT and TAT-vFLIP DED2-H1 (2H1) peptides twice for 4 days. Asterisk indicates a band of unknown identity. (E) FITC-Annexin V and 7-AAD-based flow cytometric analyses of WT and *MAVS* KO BCBL-1 cell lines treated with TAT and TAT-2H1 for 2 days. The cells were seeded at low-density (5 x10^4^ cells/ml). The percentage of total cells in each quadrant is shown. (F) CellTiter-Glo cell viability assays with the indicated cells treated with TAT and TAT-2H1 for 4 days. Data are represented as mean ± SD of two independent experiments in triplicate. (*p<0.005 and **p<0.05).

To develop a reagent to specifically disrupt the vFLIP-MAVS interaction, which could potentially provide a basis for therapeutic targeting of latently infected cells, we first wanted to identify the vFLIP region essential for MAVS binding. Co-IP assays revealed that GST-vFLIP full-length, DED1 (residues 1–90), DED2 (residues 85–188), but not GST, were co-immunoprecipitated with MAVS (Fig 9A), indicating that vFLIP contains multiple MAVS binding sites. vFLIP was predicted to contain eleven α-helices (H): H1 to H6 in DED1 and H1 to H5 in DED2 [45, 46] (Fig 9B). To further define MAVS binding sites, eleven GST-vFLIP α-helices were generated and co-transfected with Flag-MAVS into 293T cells. Co-IP assays showed that GST-DED1-H1 (1H1), GST-DED1-H6 (1H6), and GST-DED2-H1 (2H1) appeared to bind to MAVS (Fig 9B). Accordingly, MAVS-induced vFLIP stabilization was significantly inhibited by co-expression of the GST-1H1, GST-1H6, and GST-2H1 (Fig 9C). Moreover, we tested the effects of a cell-penetrating version of the vFLIP helices (TAT-1H1, TAT-1H6, and TAT-2H1; (S8A Fig)) on MAVS-induced vFLIP stabilization in 293T cells. TAT-2H1 potently inhibited (up to 97%) vFLIP expression induced by MAVS while TAT-1H1 and 1H6 inhibited the expression up to ~60% compared to the TAT peptide (S8B Fig). Importantly, TAT-2H1 abolished detectable endogenous vFLIP expression in BCBL-1 cells cultured at low density (Fig 9D) and increased cell death by more than 50% (early and late apoptotic cells) of WT and *MAVS* KO BCBL-1 cells (Fig 9E). Surprisingly, there was no significant difference of TAT-2H1-induced cell death between WT and *MAVS* KO cells: WT (clone C3), 54.55%; WT (clone C6) 53.05%; KO (clone 1A4), 56.02%; KO (clone 3B11), 58.13% (Fig 9E). Considering that vFLIP degradation was highly induced by the 2H1 peptide (Fig 9D) compared to that of MAVS-deficient BCBL-1 cells cultured at low density (Fig 2A), the 2H1 region-mediated vFLIP stabilization is likely to be achieved by some other mechanism(s) in addition to MAVS binding. Conversely, MAVS may be essential but not sufficient for vFLIP stabilization. On the other hand, cell viability assays revealed that TAT-2H1 promoted the death of other HHV-8-infected PEL cells including BC-2 and BCP-1, but not BJAB (HHV-8^–^) and AKATA (HHV-8^–^/EBV^+^) cells (Fig 9F). TAT-2H1 had no effect on other MAVS functions including IFN-β induction, and NF-κB and JNK activation (S9 Fig). Taken together, these results suggest that vFLIP peptide-mediated specific modulation of vFLIP protein stability may represent a useful therapeutic strategy for treatment of HHV-8 diseases.

### vFLIP is crucial for proliferation of HHV-8-infected cells

**Fig 10:**
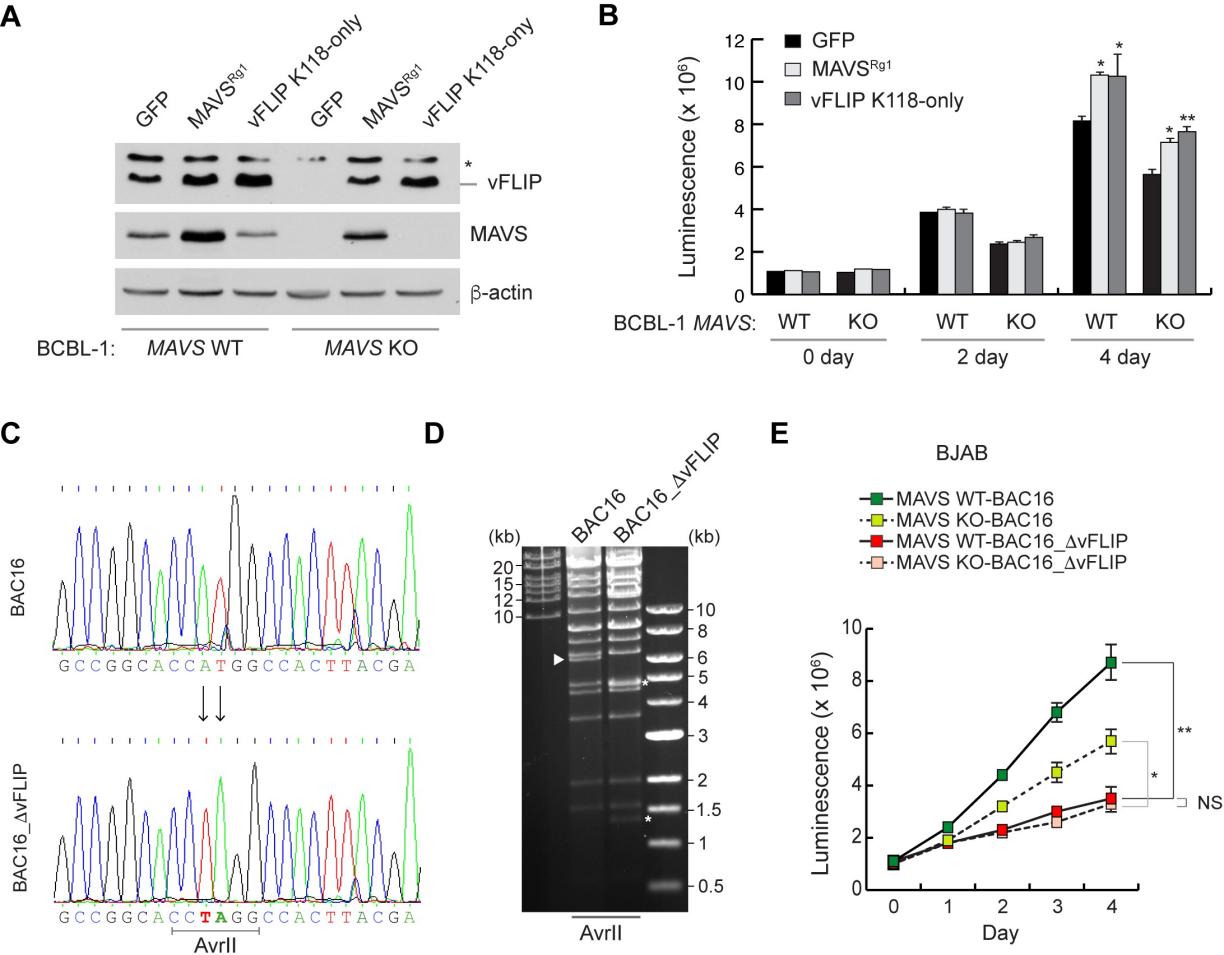
vFLIP expression is required for HHV-8 latent infection. (A) Immunoblots of the extracts from WT and *MAVS* KO BCBL-1 cells lentivirally transduced with GFP, MAVS^Rg1^, and K118-only vFLIP for 4 days. The asterisk indicates a band of unknown identity. (B) Cell viability assays with the cells seeded at 5x10^4^ cells/ml after 4 day-transduction as in (A). Data are presented as mean ± SD of triplicate samples. (*p<0.005 and **p<0.05) (C) Sequence analysis of the wild-type BAC16 and mutant BAC16_ΔvFLIP genomes. The vFLIP initiation (ATG) codon was substituted with a stop codon TAG in BAC16_ΔvFLIP, thereby creating a *de novo* AvrII restriction site. (D) Agarose gel electrophoresis of wild-type BAC16 and recombinant BAC16_ΔvFLIP genomes. DNAs were digested with AvrII and resolved on a 0.5% agarose gel stained with ethidium bromide. As predicted from AvrII restriction pattern, the 6,178 base pairs (bp) fragment (arrow head) was fragmented into two segments 1,384 and 4,794 bp (asterisks). (E) Growth curve of WT and *MAVS* KO BJAB cells infected with wild-type BAC16 and mutant BAC16_ΔvFLIP viruses. Cell viability was assessed by determining ATP levels using CellTiter-Glo for 4 days. Data are presented as mean ± SD of two independent experiments in triplicate. (*p<0.005; **p<0.05; and ns, not significant).

We next reconstituted *MAVS* KO BCBL-1 (1A4) cells with gRNA-resistant MAVS^Rg1^ and K118-only vFLIP (a stable version of vFLIP) to examine whether vFLIP is a critical factor for proliferation of BCBL-1 cells. As expected, MAVS reconstitution restored the expression of endogenous vFLIP (Fig 10A). Indeed, reconstitution of MAVS and K118-only vFLIP promoted the proliferation of *MAVS* KO BCBL-1 cells (Fig 10B). K118-only vFLIP was also functional in an NF-κB reporter assay (S10 Fig). To further delineate the functional significance of MAVS in vFLIP-mediated cell proliferation, we generated a recombinant BAC16 HHV-8 genome (BAC16_ΔvFLIP), which is defective in vFLIP expression, using Red-mediated recombination (see the Materials and Methods section for details), verified by DNA sequencing and AvrII digestion (Fig 10C and 10D), and infected WT and *MAVS* KO BJAB cells with BAC16 and BAC16_ΔvFLIP viruses that were produced from stably transfected and reactivated iSLK cells. The results demonstrated clearly, similar to the 2H1 peptide-induced cell death (Fig 9E), that genetic ablation of vFLIP expression resulted in reduced proliferation, to the same extent, of both WT and *MAVS* KO BJAB cells (Fig 10E). Therefore, death and/or impaired proliferation caused by MAVS deficiency in HHV-8-infected cells can be attributed largely to reduced vFLIP expression. In summary, our results suggest that MAVS supports the survival and proliferation of HHV-8-infected PEL cells via vFLIP stabilization.

## Discussion

MAVS is known to function as a key adaptor that connects the RLR recognition of virus infection to innate immune responses, thereby limiting virus replication and dissemination in the host. In this study, we have found that MAVS plays a novel and crucial role in promoting the survival of latently HHV-8-infected cells by stabilizing the expression of the latent protein vFLIP on peroxisomes, thereby contributing to persistent HHV-8 infection (latency). vFLIP is known to be involved in the inhibition of HHV-8 lytic reactivation [47, 48]. Thus, MAVS-mediated vFLIP stabilization is likely also to contribute to suppression of HHV-8 lytic replication; however, TAT-2H1-mediated destabilization of vFLIP could not promote lytic gene expression and productive replication in BCBL-1 cells (S11 Fig). Therefore, our results suggest that MAVS-mediated vFLIP stabilization contributes mainly to the survival of HHV-8 latently infected cells.

A recent Integrated Systems Biology analysis revealed that peroxisome biogenesis is enhanced in HHV-8 latently-infected endothelial cells along with increased expression of several peroxisomal proteins including PEX19 [49], suggesting a contribution of peroxisomes to the successful maintenance of HHV-8 latency. Here we demonstrated that vFLIP can be localized to peroxisomes in a PEX19-dependent manner where it is stabilized by MAVS. Furthermore, vFLIP expression was significantly reduced in PEX19-deficient cells, which are depleted of peroxisomal structures. Importantly, peroxisome-targeted vFLIP (vFLIP-Pex) retained NEMO/IKKγ binding activity and NF-κB activation. Thus, it is likely that an increase in PEX19 expression and peroxisome biogenesis in HHV-8 latently-infected cells contributes to MAVS-mediated stabilization of vFLIP and subsequent maintenance of latency.

Our data indicate that vFLIP targeting to peroxisomes appears to be mediated by PEX19. There are different models ascribing PEX19-dependent trafficking of the peroxisomal membrane proteins (PMPs) during peroxisome biogenesis. In the peroxisomal growth and division model, PEX19 serves as a chaperone that delivers nascent mPTS-containing PMPs from free cytosolic ribosomes to the peroxisomal membrane [41]. In the *de novo* peroxisomal biogenesis model, PEX19 shuttles the PMPs together with the membrane import receptors PEX3 and PEX16 through the generation of pre-peroxisomal vesicles that originate from the endoplasmic reticulum (ER) and mitochondria [50, 51]. Our imaging studies detected vFLIP with a distinct speckled pattern in the cytoplasm that in part co-localized with and was juxtaposed to the peroxisomes, implying that vFLIP may be targeted to peroxisomes via the pre-peroxisomal vesicles rather than direct delivery from the cytosol. However, there is no evidence that vFLIP is localized on the membranes of the ER or mitochondria and contains a secondary structure required for membrane targeting. Using software tools such as TMbase and the Dense Alignment Surface method, we predicted a putative transmembrane helix of vFLIP encompassing residues 20 to 40, which is positioned adjacent to the mPTS of vFLIP; however, it needs to be verified experimentally that the predicted region is indeed required for membrane targeting. In fact, cFLIP-L was reported to be localized at the ER and mitochondria-associated ER membranes [52], despite the lack of a transmembrane domain. Thus, we speculate that vFLIP might be localized to the ER or mitochondria via its putative transmembrane domain or protein-protein interactions and then delivered to peroxisomes through PEX19-dependent pre-peroxisomal vesicles.

vFLIP is a critical viral oncoprotein that is essential for the survival of HHV-8-associated tumors. Thus, there has been considerable interest in manipulating the function and stability of vFLIP for the development of drugs to treat HHV-8-related tumors. For example, two independent groups reported that the purine scaffold HSP90 inhibitors, PU-H71 and BIIB021, suppressed tumor growth in mice xenografted with HHV-8^+^ PEL cells, potentially by promoting vFLIP degradation and inhibiting vFLIP-mediated NF-κB activation [33, 53]. Another group showed that vFLIP-derived peptides that inhibit interaction of vFLIP and autophagy-related protein 3 (ATG3) suppress tumor formation in NOD/SCID mice xenografted with PEL cells [32]. Nonetheless, targeted ablation of vFLIP protein may represent a more efficacious approach in the treatment of HHV-8-related malignancies. We found that the vFLIP-derived peptide 2H1, that inhibits vFLIP interaction with and stabilization by MAVS, could abolish detectable expression of vFLIP in HHV-8-infected PEL cells and selectively induce the death of HHV-8^+^ PEL cells, indicating the potential of this approach to treat HHV-8 diseases.

In summary, the data presented here identify molecular mechanisms and functional significance of MAVS-mediated vFLIP stabilization on peroxisomes. To our knowledge, this is the first report of MAVS and peroxisome function in the regulation of viral oncogenic protein stability and therefore our data have revealed a novel mechanism underlying the regulation of a viral protein essential for HHV-8 latency and associated pathogenesis.

## Materials and methods

### Cell culture

BCBL-1 TRE-RTA (a gift from Dr. Jae U. Jung), BCBL-1, BC-2 and BCP-1 (ATCC), and AKATA and BJAB (a gift from Dr. Richard Ambinder) cells were cultured in RPMI 1640 supplemented with 15% fetal bovine serum (FBS), antibiotics including streptomycin and penicillin, and plasmocin prophylactic (Invivogen). 293, 293T, 293A (Thermo Fisher Scientific), and iSLK and iSLK-BAC16 (a gift from Dr. Jae U. Jung) cells were cultured in DMEM supplemented with 10% FBS and antibiotics. Transient and stable transfections with plasmids were performed using GenJet version II (SignaGen Laboratories) following the manufacturer’s instruction. For generation of MAVS-deficient cells, BCBL-1 TRE/RTA, AKATA, and BJAB cells were lentivirally transduced with MAVS gRNAs in the presence of 5 μg/ml polybrene overnight and puromycin-resistant cells were selected, transferred individually into a 96-well plate, and expanded in the presence of puromycin for an additional month. MAVS deficiency in each clone was verified by immunoblotting using anti-MAVS antibody. For *PEX19* KO 293A cells, cells were stably transfected with the lentiCRISPR V2 vector encoding PEX19 gRNA using GenJet reagent. After puromycin selection of single clones, PEX19 deficiency was verified by immunoblotting using anti-PEX19 antibody. BJAB cells were infected by BAC16 viruses, which were isolated from iSLK-BAC16 and iSLK-BAC16_ΔvFLIP cells reactivated by treatment with 1 μg/ml doxycycline and 1 mM sodium butyrate for 3 days, by spinoculation at 800 x g for 30 min at room temperature and then cultured in the presence of hygromycin (500 μg/ml) for 3 days. Viable BAC16 virus-infected BJAB cells were isolated using Histopaque-1077 medium.

### DNA manipulation

All polymerase chain reaction (PCR) amplification and site-directed mutagenesis including point and deletion mutations were performed using Platinum *Pfx* DNA polymerase (Thermo Fisher Scientific). Subcloning of an open reading frame (ORF) and its derivatives into expression vectors including pICE (Addgene) was performed using appropriate restriction enzyme sites (S2 Table). For construction of vFLIP-Pex, the DNA fragment encompassing PEX13 residues 145–233 was amplified from MAVS-Pex and fused to the C-terminal end of vFLIP by overlap extension PCR, and the chimeric construct was cloned into pICE_V5 vector (S2 Table). The small guide RNAs (gRNAs) with target sequences specific for MAVS and PEX19 (S1 Table) were cloned into lentiCRISPR V2-Puro vector (Addgene). The MAVS ORF (MAVS^Rg1^) resistant to the gRNA1 was generated by site-directed mutagenesis using degenerated primers (S1 Table).

### Mutagenesis of HHV-8 bacmid BAC16

The BAC16 genome was edited using a two-step seamless Red recombination in the context of *E*. *coli* strain GS1783 (a kind gift from Greg Smith) as previously described [54]. Briefly, PCR amplification was performed to generate a linear DNA fragment containing a kanamycin resistance expression cassette, an I-SceI restriction enzyme site, and flanking sequences derived from HHV-8 genomic DNA, each of which includes a 43-bp copy of a duplication. The mutated codon of the translation initiation codon of vFLIP (ATG > TAG) was positioned in the middle of the duplication. This fragment was purified and then electroporated into GS1783 cells harboring BAC16 and transiently expressing *gam*, *bet*, and *exo*, which are expressed in a temperature-inducible manner from the lambda Red operon in the GS1783 chromosome. The integrated Kan^R^/I-SceI cassette was cleaved by I-SceI enzyme that was inducibly expressed by treatment with 1% arabinose, resulting in a transiently linearized BAC16. A second Red-mediated recombination between the duplicated sequences results in recircularization of the BAC DNA and seamless loss of the Kan^R^/I-SceI cassette. Kanamycin-sensitive colonies were selected via replica plating. The BAC DNAs were purified using the NucleoBond BAC 100 kit (Clontech). The recombination area was amplified by PCR and the mutation was verified by DNA sequencing of the PCR amplicon. Gross genomic integrity was verified using digestion with AvrII restriction enzyme, which additionally recognizes the mutated codon of vFLIP, and agarose gel analysis of digestion profiles.

### Immunoblotting, immunoprecipitation, and immunostaining

Antibodies used in the immunological assays including immunoblotting, immunoprecipitation, and immunostaining are listed in S3 Table. For the preparation of total cell extracts, cells were resuspended in RIPA buffer (50 mM Tris [pH 7.4], 150 mM NaCl, 1% Igepal CA-630, and 0.25% deoxycholate) containing protease inhibitor cocktail and protein phosphatase inhibitors including 10 mM NaF and 5 mM Na_3_VO_4_ and sonicated using Bioruptor (Diagenode, Denville, NJ) for 5 min in ice water at a high-power setting (320 W). For immunoblotting, total cell extracts were separated by SDS-PAGE, transferred to nitrocellulose or PVDF membranes, and immunoblotted with appropriate antibodies diluted in SuperBlock (phosphate-buffered saline [PBS]) blocking buffer (Thermo Fisher Scientific). The immunoreactive bands were detected by Clarity Western ECL reagents (Bio-Rad) on an ECL film. For immunoprecipitation (IP), total cell extracts were incubated with specific primary antibody at 4°C overnight and incubated with protein G-agarose beads (Cell Signaling Technology) for an additional 3 h. Immunoprecipitates were washed with RIPA buffer, followed by elution of bound proteins with 1.5× SDS sample buffer or 3× Flag peptide (Sigma, St. Louis, MO). For the IP of Flag- and V5-tagged proteins, anti-DYKDDDDK tag (L5) affinity gel (BioLegend) and anti-V5-agarose (Sigma) were used, respectively. To avoid detection of IgG used in IP, Clean-Blot IP detection reagent (Thermo Fisher Scientific) was used. For ubiquitination assays, an extra wash was performed using RIPA buffer supplemented with 1 M urea before elution. For immunostaining, cells grown on a coverslip (and transfected) were fixed in 4% formaldehyde prepared in PBS and permeabilized in 0.5% Triton X-100 prepared in Dulbecco’s PBS (DPBS). Following incubation with 3% bovine serum albumin in DPBS for 1 h at room temperature, coverslips were incubated with primary antibodies, washed with PBS, and then incubated with appropriate fluorescent dye-conjugated secondary antibodies. Stained cells were imaged on the Zeiss 700 confocal laser scanning microscope with a 40X oil-corrected objective and Zen software.

### Virus manipulation and transduction

For lentivirus production, 293T cells were co-transfected with the lentiviral transfer vector together with the packaging plasmid psPAX2 and the vesicular stomatitis virus G protein expression plasmid pVSV-G at a ratio of 5:4:1 using GenJet (Ver. II) transfection reagent (SignaGen). Two days later, virus was collected from the medium by ultracentrifugation in an SW28 rotor at 25,000 rpm for 2 h at 4°C. Virus pellets were resuspended in an appropriate volume of PBS to achieve 100x concentration. The transduction unit of lentiviruses was determined in 293T cells in the presence of appropriate antibiotics. To produce infectious BAC16 viruses, iSLK-BAC16 cells were reactivated with 1 μg/ml doxycycline and 1 mM sodium butyrate for 3 days. Infectious titer of BAC16 virus in the culture supernatant was determined by spinoculation of 293A cells and 1 day later GFP-positive cells were quantified using flow cytometry. BJAB cells were infected with 1 infectious unit of BAC16 by spinoculation at 800 x g for 30 min at room temperature. For lentiviral transduction of GFP, MAVS rgRNA1, and K118-only vFLIP (in pDUET110) into BCBL-1 cells, cells were incubated with 1 transduction unit (TU) in the presence of 5 μg/ml polybrene for 1 day and washed in complete RPMI 1640 media, and further cultured for cell viability assays and immunoblotting assays.

### GST-pull down assays

Recombinant GST and GST-fusion proteins were expressed in Rosetta cells and cleared lysates containing 1 μg GST and GST-fusion proteins were incubated with 20 μl bed volume of washed glutathione sepharose-4B beads for 1 h at room temperature. After washing in binding buffer, the protein-bead complexes were incubated with 1 μg purified recombinant human MAVS protein (ProSpec, Pro-1351) or cell extracts containing HA-PEX19 protein at 4°C overnight, washed in binding buffer four times, and separated on SDS-PAGE and subjected to immunoblotting.

### Reporter assay

293T cells were plated in triplicate and the next day transfected with 100 ng of reporter plasmids and 400 ng of expression plasmids using GenJet Ver. II reagent. pRL-TK vector (5 ng) was included in the transfection mixture to monitor the efficiency of the transfections. Cells were extracted with passive lysis buffer (Promega), and Firefly and *Renilla* luciferase activities were determined using a Dual-Luciferase Reporter Assay kit (Promega). The same cell extracts were used for immunoblotting analysis.

### Cell death and viability assays

Dead cells present prior to experimentation were removed using Histopaque-1077 (Sigma) or Dead Cell Removal kit (Miltenyl Biotec). Freshly isolated intact cells were cultured at different cell densities (5x10^4^ and 2x10^5^ cells/ml) or treated as indicated in the Figure Legends. Cells were then stained with FITC-annexin V and 7-amino-actinomycin D (7-AAD) as described by the manufacturer (BioLegend). Annexin V and 7-AAD were detected in FL1 and FL3, respectively, using BD FACSCalibur flow cytometry. For cell viability assays, the CellTiter-Glo^®^ Luminescence kit (Promega) was used. In brief, 50 μl of cell culture in triplicate was transferred to a 96-well white plate and 50 μl of the CellTiter Glo^®^ reagents was added to the culture. After vigorous shaking at RT for 2 min, the mixtures were incubated for 30 min at room temperature and their luminescence was measured using the GloMax Microplate Luminometer (Promega).

### Real time-quantitative PCR (RT-qPCR) analysis

Total RNAs were isolated from latent and reactivated BCBL-1 cells or transfected cells using the RNeasy mini kit (Qiagen). First-strand cDNA was synthesized from 1 μg of total RNA using SuperScript II reverse transcriptase (Invitrogen) with random hexamers. RT-qPCR was performed using an ABI Prism 7500 system (Applied Biosystem) with the RT^2^Real-Time SYBR green/ROX master mix (Qiagen). Reactions were performed in a total volume of 25 μl and contained 50 ng of reverse-transcribed RNA (based on the initial RNA concentration) and gene-specific primers. PCR conditions included an initial incubation step of 2 min at 50°C and an enzyme heat activation step of 10 min at 95°C, followed by 40 cycles of 15 seconds at 95°C for denaturing and 1 min at 60°C for annealing and extension.

### HHV-8 replication assay

For determination of encapsidated HHV-8 genome copy number from the culture supernatant, viral DNA was purified using Quick-DNA™ Viral Kit (Zymo Research) following pretreatment of virus suspension with DNase I (New England BioLabs) at 37°C overnight. RT-qPCR was performed using ABI PRISM 7500 system as described above with the LANA primers (S1 Table).

### Reagents

The TAT and TAT-vFLIP peptides were custom-synthesized with purity of more than 95% (Biomatik). Rapamycin and MG132 were purchased from Cell Signaling Technology. PU-H71 was purchased from Selleckchem. EBSS was purchased from Thermo Fisher Scientific. Recombinant TRAIL was purchased from BioLegend. Staurosporin, CCCP, 3-methyladenine, rotenone, bafilomycin A1, chloroquine, and cycloheximide were purchased from Sigma.

### Quantification and statistical analysis

Band intensity of immunoblots was determined using ImageJ software. Statistical parameters including statistical analysis, statistical significance, and p value are stated in the Figure legends and Supplemental Figure legends. Statistical analyses were performed using Synergy software (KaleidaGraph). For statistical comparison of cell viability assays, standard paired t-test was used. A value of p < 0.05 was considered significant.

## Supporting information

S1 Fig Real time-quantitative PCR (RT-qPCR) analysis of expression of HHV-8 lytic genes and IFNs. Total RNAs isolated from wild-type (WT, clone C6) and *MAVS* knockout (KO, clone 1A4) BCBL-1/TRE-RTA cells treated with 1 μg/ml doxycycline for 0, 1, and 2 days were reverse transcribed and used in RT-qPCR. The relative expression of each gene, including (A) MIR-2 (K5), (B) K8.1, (C) IFN-α1, (D) IFN-γ, (E) IFN-β1, (F) IFN-β2 (IL6), and (G) IFN-λ1, was normalized first with the house keeping gene, β-actin, and then divided by the average expression level at day 0. The HHV-8 lytic genes analyzed include immediate early gene MIR-2 (K5) and late gene K8.1. Data are represented as mean ± SD of triplicate samples.
(TIF)

S2 Fig Characterization of cell death and HHV-8 productive replication in MAVS-deficient BCBL-1 cells. (A) Flow cytometry analysis using annexin V-FITC and 7-AAD in WT and *MAVS* KO BCBL-1 (1A4) cells untreated and treated with 10 μM zVAD-fmk for 1 day. The cells were seeded at 2x10^5^ cells/ml. (B) HHV-8 productive replication assay. HHV-8 viral genomes were purified from the culture supernatants of WT (C6) and *MAVS* KO (1A4 and 3B11) BCBL-1 cells grown under high-density culture for 2 days and subjected to quantitative PCR to determine the copy number of the viral genome. Data are represented as mean ± SD of triplicate samples. (C) The cells were incubated in EBSS for 6 h or treated with rapamycin (Rapa), 50 ng/ml TNF-related apoptosis-inducing ligand (TRAIL), 100 nM staurosporine (STS), 10 μM carbonyl cyanide 3-chlorophenylhydrazone (CCCP), and 5 μM rotenone (Rot) in complete media for 1 day. Cell viability was assessed by using CellTiter-Glo^®^. Data are represented as mean ± SD of two independent experiments in triplicate. (*p<0.005 and **p<0.05).
(TIF)

S3 Fig p62/SQSTM1 expression in WT and *MAVS* KO BJAB and AKATA cells. Immunoblotting was performed with extracts derived from the BJAB and AKATA cells cultured at different densities, low (5x10^4^ cells/ml) and high (2x10^5^ cells/ml), for 2 days.
(TIF)

S4 Fig Effect of epitope tagging on basal and MAVS-induced vFLIP stability. Extracts from 293T cells transfected with the indicated epitope tagged and non-tagged vFLIPs together with or without Flag-MAVS, for 24 h were separated by SDS-PAGE and immunoblotted with anti-vFLIP, Flag, and β-actin antibodies.
(TIF)

S5 Fig Real time-qPCR analysis of V5-vFLIP expression in TRAF6-cotransfected cells. Total RNAs were isolated from WT and *MAVS* KO 293T cells co-transfected with pICE_V5-vFLIP plasmid together with the indicated amounts of Flag-TRAF6 plasmid for 24 h and subjected to real time-qPCR. The relative mRNA expression of V5-vFLIP normalized to 18S RNA was determined by comparison to control (WT cells transfected with V5-vFLIP without TRAF6) and depicted in the column graph. Data are represented as mean ± SD of triplicate samples. “NS” indicates not significant (p>0.1).
(TIF)

S6 Fig TRAF6 partially localizes to peroxisomes in a MAVS-dependent manner. Triple immunostaining with antibodies to Flag (TRAF6), MAVS, and PMP70 in WT and *MAVS* KO 293T cells transfected with Flag-TRAF6 together with or without MAVS-Pex. Fluorescent images were merged with an image of DAPI. The inset boxes in the merged images were zoomed in to the right side of the images. Yellow dots indicate localization of TRAF6 to peroxisomes and white dots indicate co-localization of TRAF6 and MAVS on peroxisomes. Scale bar indicates 10 μm.
(TIF)

S7 Fig Peroxisomes are required for MAVS-induced vFLIP stabilization. Triple immunostaining with antibodies to Flag (MAVS), V5, and PMP70 in WT and *PEX19* KO 293A cells transfected with V5-vFLIP WT or mPTS^X^ together with Flag-MAVS, Flag-MAVS-Mito, and Flag-MAVS-Pex. Fluorescent images were merged with an image of DAPI. The inset boxes in the merged images were zoomed in at the bottom of the figure. Yellow dots indicate localization of vFLIP to peroxisomes and white dots indicate co-localization of vFLIP and MAVS on peroxisomes. V5-vFLIP was barely detected in *PEX19* KO cells, and V5-vFLIP mPTS^X^ was barely detected in WT and *PEX19* KO cells. Scale bar indicates 20 μm.
(TIF)

S8 Fig The effect of cell-penetrating versions of vFLIP-derived peptides on MAVS-induced vFLIP stabilization. (A) Sequences of TAT and TAT-fused vFLIP peptides. (B) Immunoblotting with extracts of 293A cells co-transfected with V5-vFLIP and empty (–MAVS) or Flag-MAVS (+ MAVS) vectors and then treated with the peptides for 1 day.
(TIF)

S9 Fig The effect of the vFLIP peptide 2H1 on MAVS-induced antiviral responses. (A-B) Reporter assays in 293T cells transfected with empty (–MAVS) or Flag-MAVS (+ MAVS) vectors along with IFN-β-Luc (A) or NF-κB-Luc (B) reporter in the presence of TAT and TAT-2H1 peptides for 1 day. Data are presented as mean ± SD of triplicate samples. “NS” indicates not significant. (C) Immunoblots of extracts of 293T cells transfected with empty vector or MAVS plasmid in the presence of TAT and TAT-2H1 peptides for 1 day.
(TIF)

S10 Fig K118-only vFLIP is functional. Reporter assay was performed in 293T cells transfected with empty vector, vFLIP, or K118-only vFLIP vectors along with NF-κB-Luc (B) reporter plasmid for 1 day. Data are presented as mean ± SD of triplicate samples.
(TIF)

S11 Fig vFLIP degradation by the 2H1 peptide does not affect HHV-8 reactivation. (A) RT-qPCR analysis of encapsidated HHV-8 genome in the supernatants of BCBL-1 culture. Cells were left untreated or treated with 20 ng/ml of phorbol myristate acetate (PMA), a lytic inducer, together with 10 μM TAT or TAT-2H1 peptides for 3 days. The viral genome copy number was determined using a standard curve of BAC16 DNA; fold change (relative copy number) was calculated by dividing the values of the samples with that of control (TAT and no PMA). Data are presented as mean ± SD of triplicate samples. (B) Immunoblotting analysis of extracts of the BCBL-1 cells treated as in (A).
(TIF)

S1 Table Oligonucleotides used in the study. (PDF)

S2 Table Plasmids used in the study. (PDF)

S3 Table Antibodies used in the study. (PDF)
