# Supplementary figures and images for: Peroxisomes support human herpesvirus 8 latency by stabilizing the viral oncogenic protein vFLIP via the MAVS-TRAF complex
Source: PLoS Pathog. 2018 May 10;14(5):e1007058. doi: 10.1371/journal.ppat.1007058 (PMC5963799; doi:10.1371/journal.ppat.1007058)

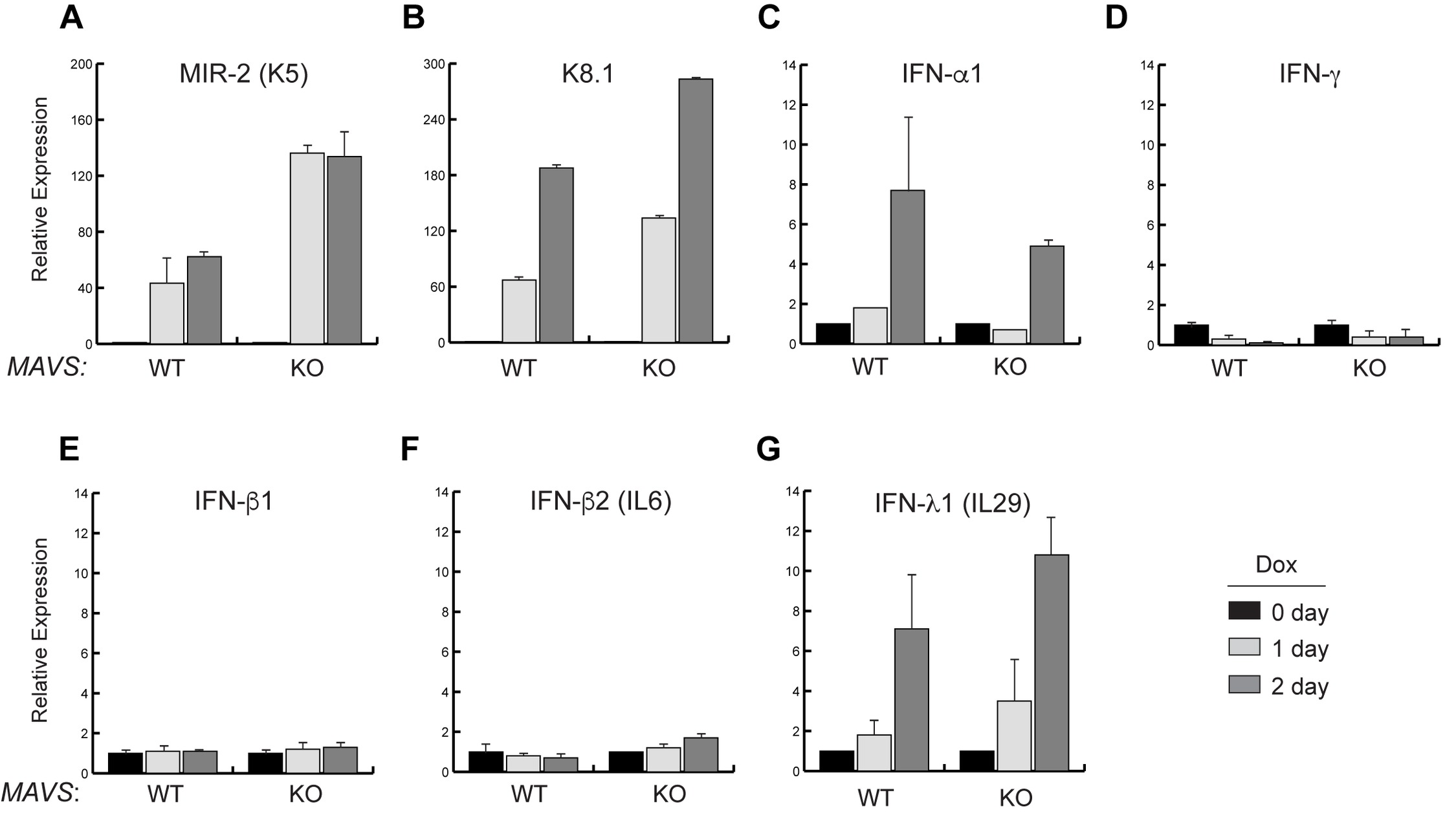

Supplement: S1 Fig — Total RNAs isolated from wild-type (WT, clone C6) and MAVS knockout (KO, clone 1A4) BCBL-1/TRE-RTA cells treated with 1 μg/ml doxycycline for 0, 1, and 2 days were reverse transcribed and used in RT-qPCR. The relative expression of each gene, including (A) MIR-2 (K5), (B) K8.1, (C) IFN-α1, (D) IFN-γ, (E) IFN-β1, (F) IFN-β2 (IL6), and (G) IFN-λ1, was normalized first with the house keeping gene, β-actin, and then divided by the average expression level at day 0. The HHV-8 lytic genes analyzed include immediate early gene MIR-2 (K5) and late gene K8.1. Data are represented as mean ± SD of triplicate samples. (TIF) [file ppat.1007058.s001.tif]

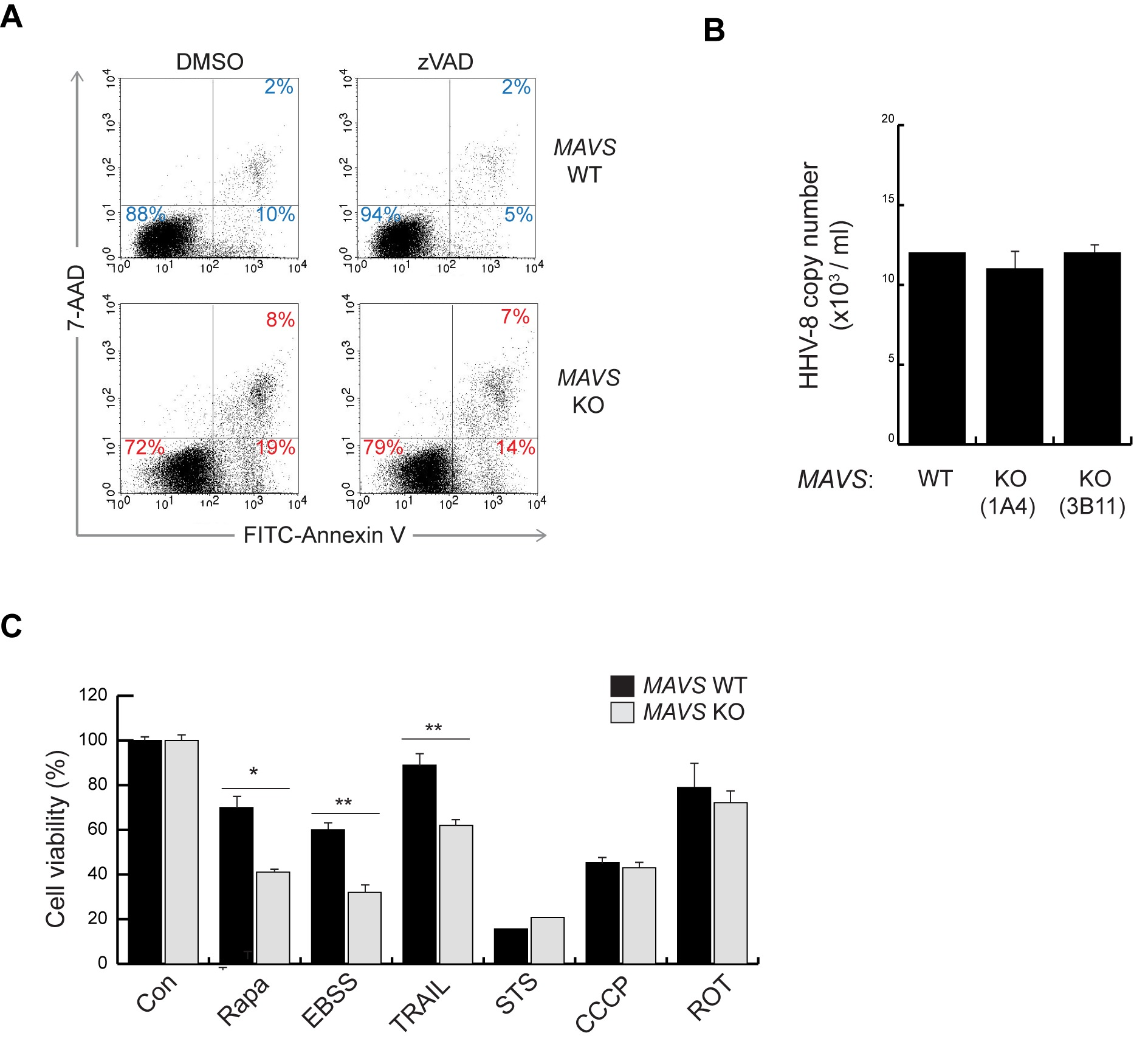

Supplement: S2 Fig — (A) Flow cytometry analysis using annexin V-FITC and 7-AAD in WT and MAVS KO BCBL-1 (1A4) cells untreated and treated with 10 μM zVAD-fmk for 1 day. The cells were seeded at 2x10^5^ cells/ml. (B) HHV-8 productive replication assay. HHV-8 viral genomes were purified from the culture supernatants of WT (C6) and MAVS KO (1A4 and 3B11) BCBL-1 cells grown under high-density culture for 2 days and subjected to quantitative PCR to determine the copy number of the viral genome. Data are represented as mean ± SD of triplicate samples. (C) The cells were incubated in EBSS for 6 h or treated with rapamycin (Rapa), 50 ng/ml TNF-related apoptosis-inducing ligand (TRAIL), 100 nM staurosporine (STS), 10 μM carbonyl cyanide 3-chlorophenylhydrazone (CCCP), and 5 μM rotenone (Rot) in complete media for 1 day. Cell viability was assessed by using CellTiter-Glo®. Data are represented as mean ± SD of two independent experiments in triplicate. (*p<0.005 and **p<0.05). (TIF) [file ppat.1007058.s002.tif]

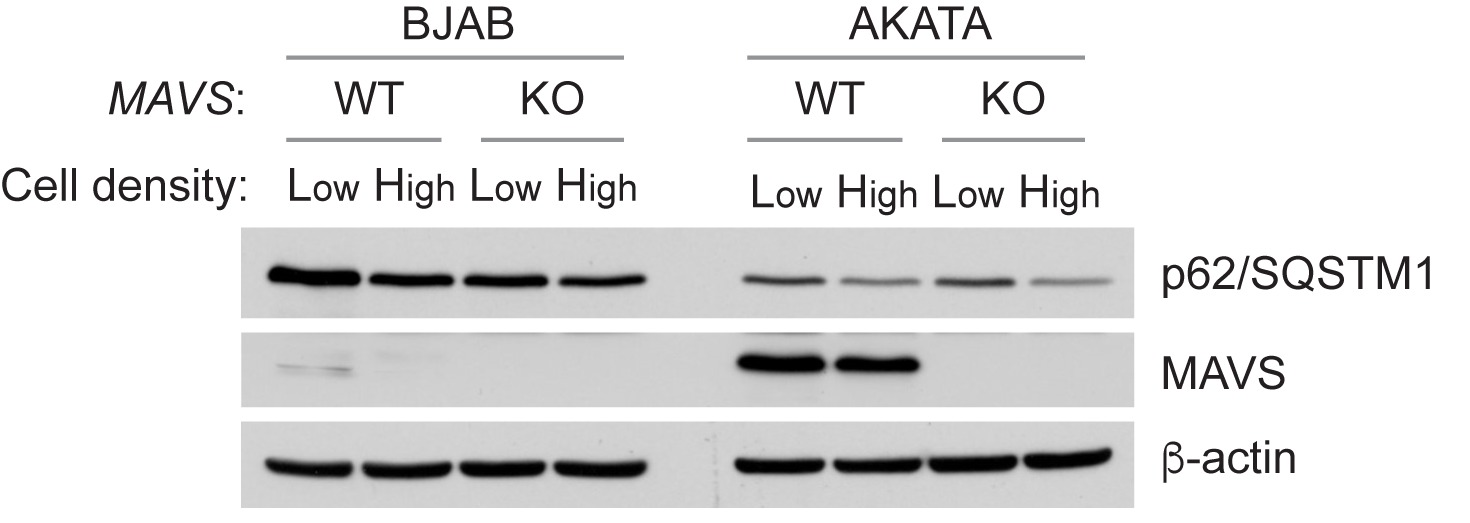

Supplement: S3 Fig — Immunoblotting was performed with extracts derived from the BJAB and AKATA cells cultured at different densities, low (5x10^4^ cells/ml) and high (2x10^5^ cells/ml), for 2 days. (TIF) [file ppat.1007058.s003.tif]

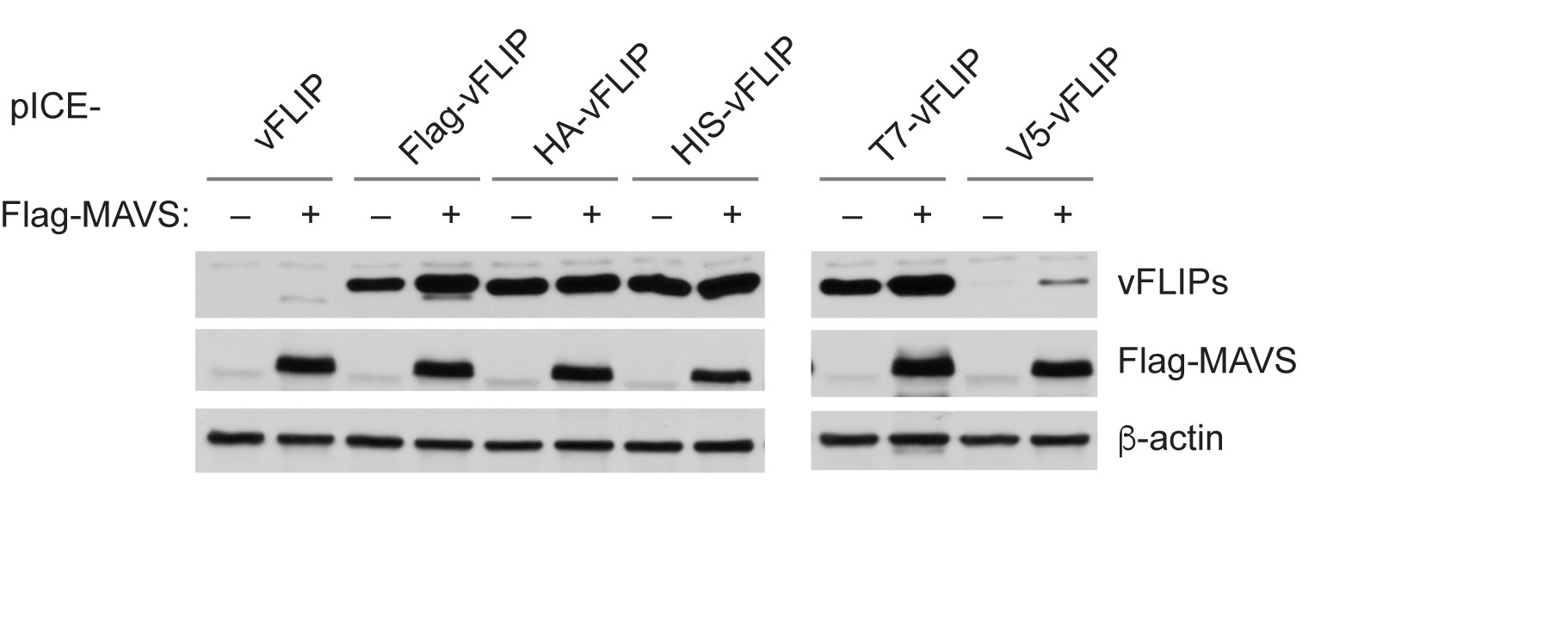

Supplement: S4 Fig — Extracts from 293T cells transfected with the indicated epitope tagged and non-tagged vFLIPs together with or without Flag-MAVS, for 24 h were separated by SDS-PAGE and immunoblotted with anti-vFLIP, Flag, and β-actin antibodies. (TIF) [file ppat.1007058.s004.tif]

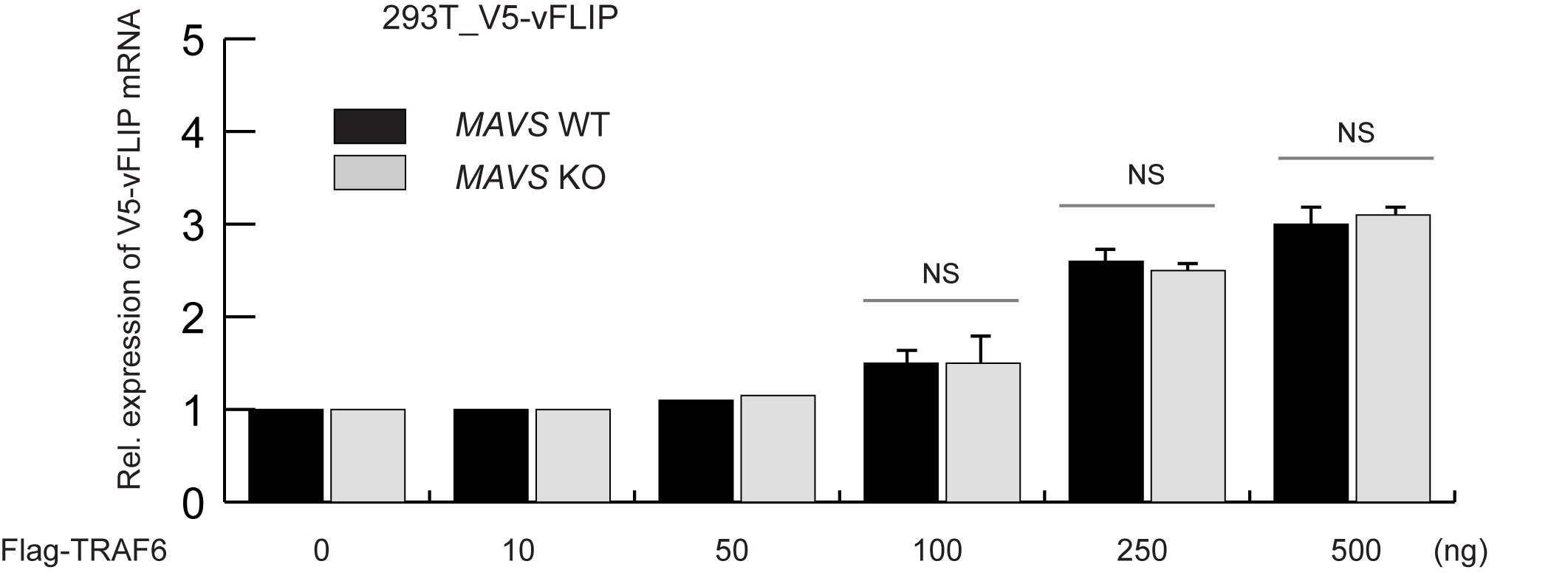

Supplement: S5 Fig — Total RNAs were isolated from WT and MAVS KO 293T cells co-transfected with pICE_V5-vFLIP plasmid together with the indicated amounts of Flag-TRAF6 plasmid for 24 h and subjected to real time-qPCR. The relative mRNA expression of V5-vFLIP normalized to 18S RNA was determined by comparison to control (WT cells transfected with V5-vFLIP without TRAF6) and depicted in the column graph. Data are represented as mean ± SD of triplicate samples. “NS” indicates not significant (p>0.1). (TIF) [file ppat.1007058.s005.tif]

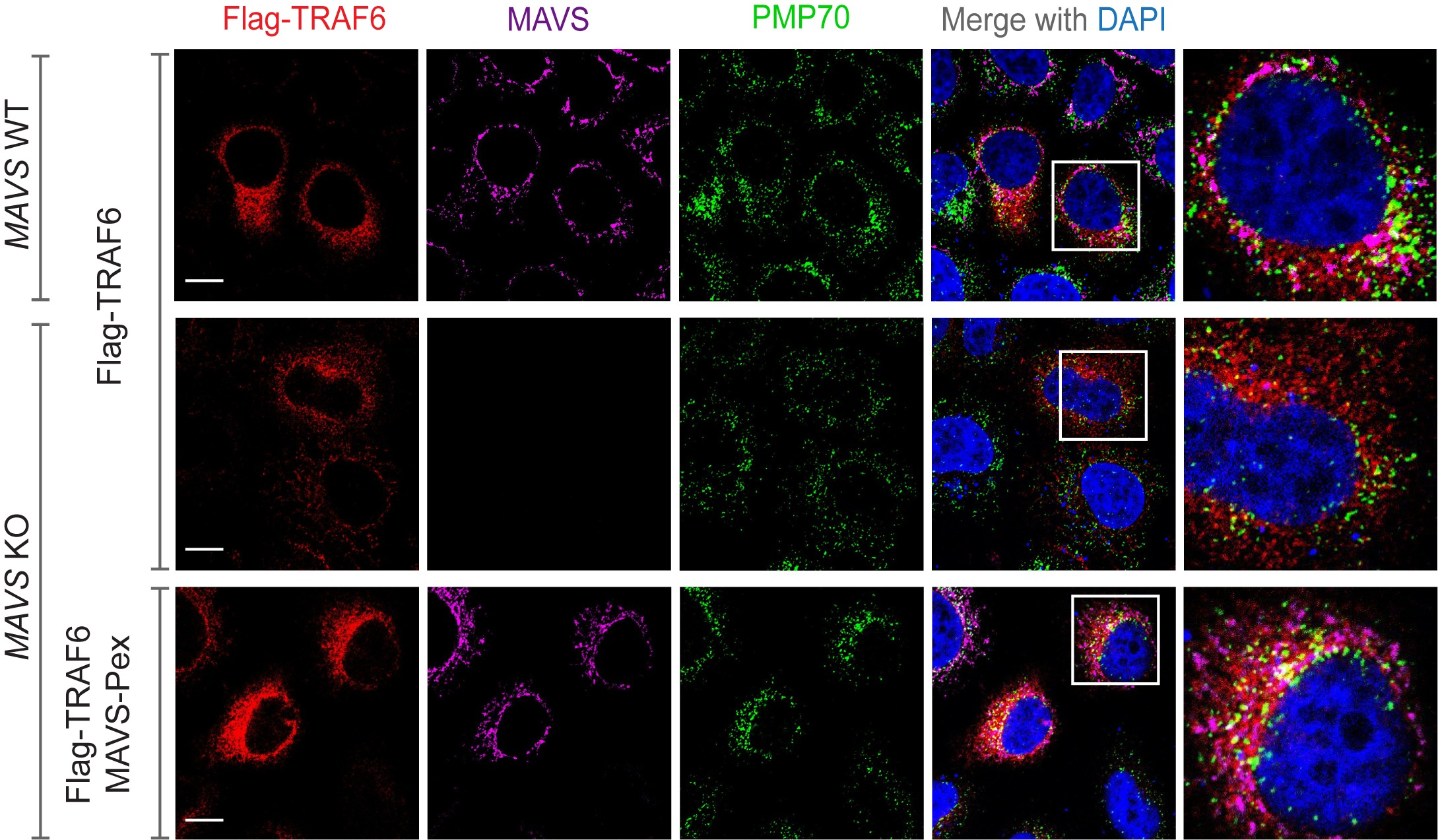

Supplement: S6 Fig — Triple immunostaining with antibodies to Flag (TRAF6), MAVS, and PMP70 in WT and MAVS KO 293T cells transfected with Flag-TRAF6 together with or without MAVS-Pex. Fluorescent images were merged with an image of DAPI. The inset boxes in the merged images were zoomed in to the right side of the images. Yellow dots indicate localization of TRAF6 to peroxisomes and white dots indicate co-localization of TRAF6 and MAVS on peroxisomes. Scale bar indicates 10 μm. (TIF) [file ppat.1007058.s006.tif]

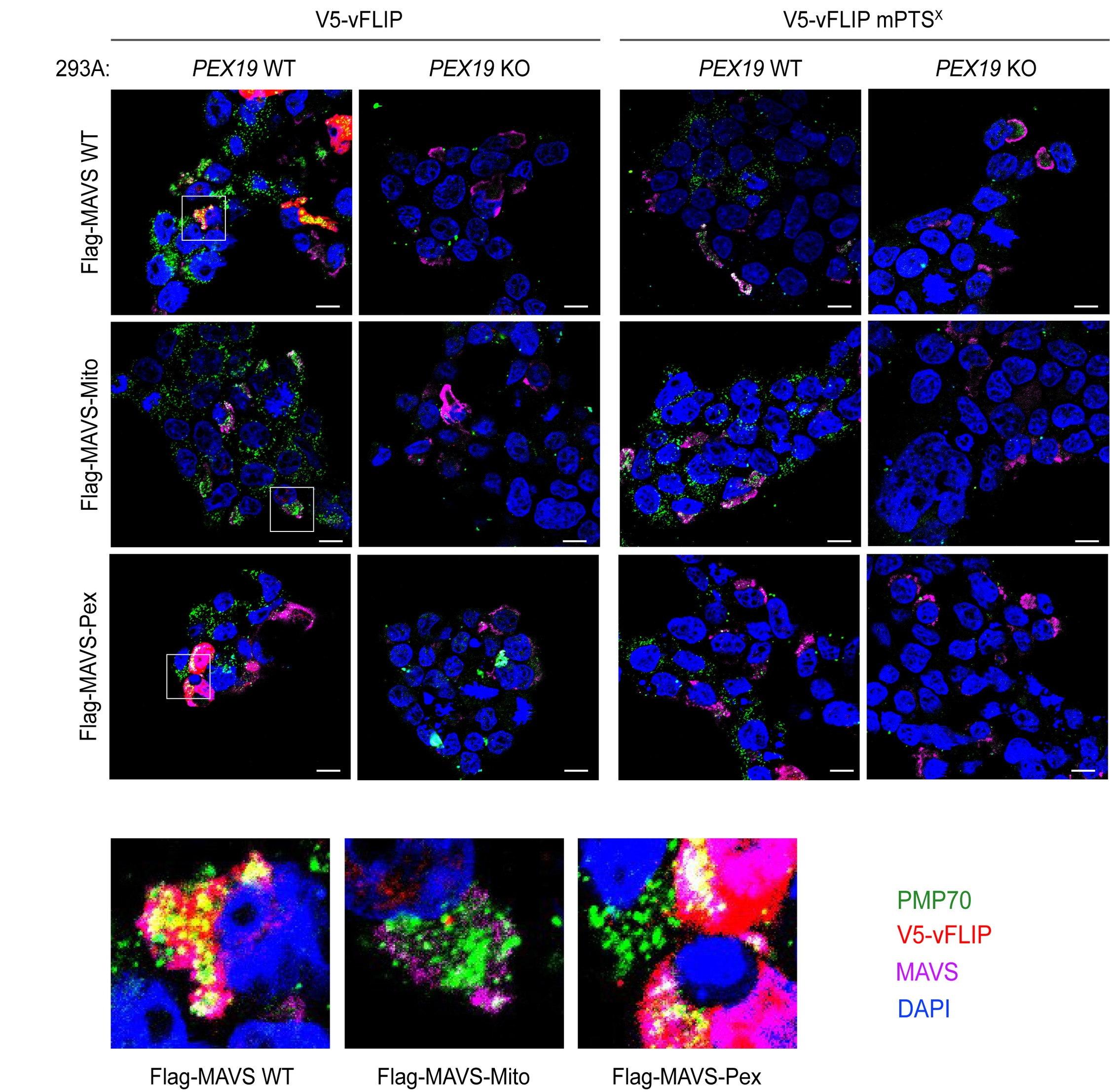

Supplement: S7 Fig — Triple immunostaining with antibodies to Flag (MAVS), V5, and PMP70 in WT and PEX19 KO 293A cells transfected with V5-vFLIP WT or mPTSX together with Flag-MAVS, Flag-MAVS-Mito, and Flag-MAVS-Pex. Fluorescent images were merged with an image of DAPI. The inset boxes in the merged images were zoomed in at the bottom of the figure. Yellow dots indicate localization of vFLIP to peroxisomes and white dots indicate co-localization of vFLIP and MAVS on peroxisomes. V5-vFLIP was barely detected in PEX19 KO cells, and V5-vFLIP mPTSX was barely detected in WT and PEX19 KO cells. Scale bar indicates 20 μm. (TIF) [file ppat.1007058.s007.tif]

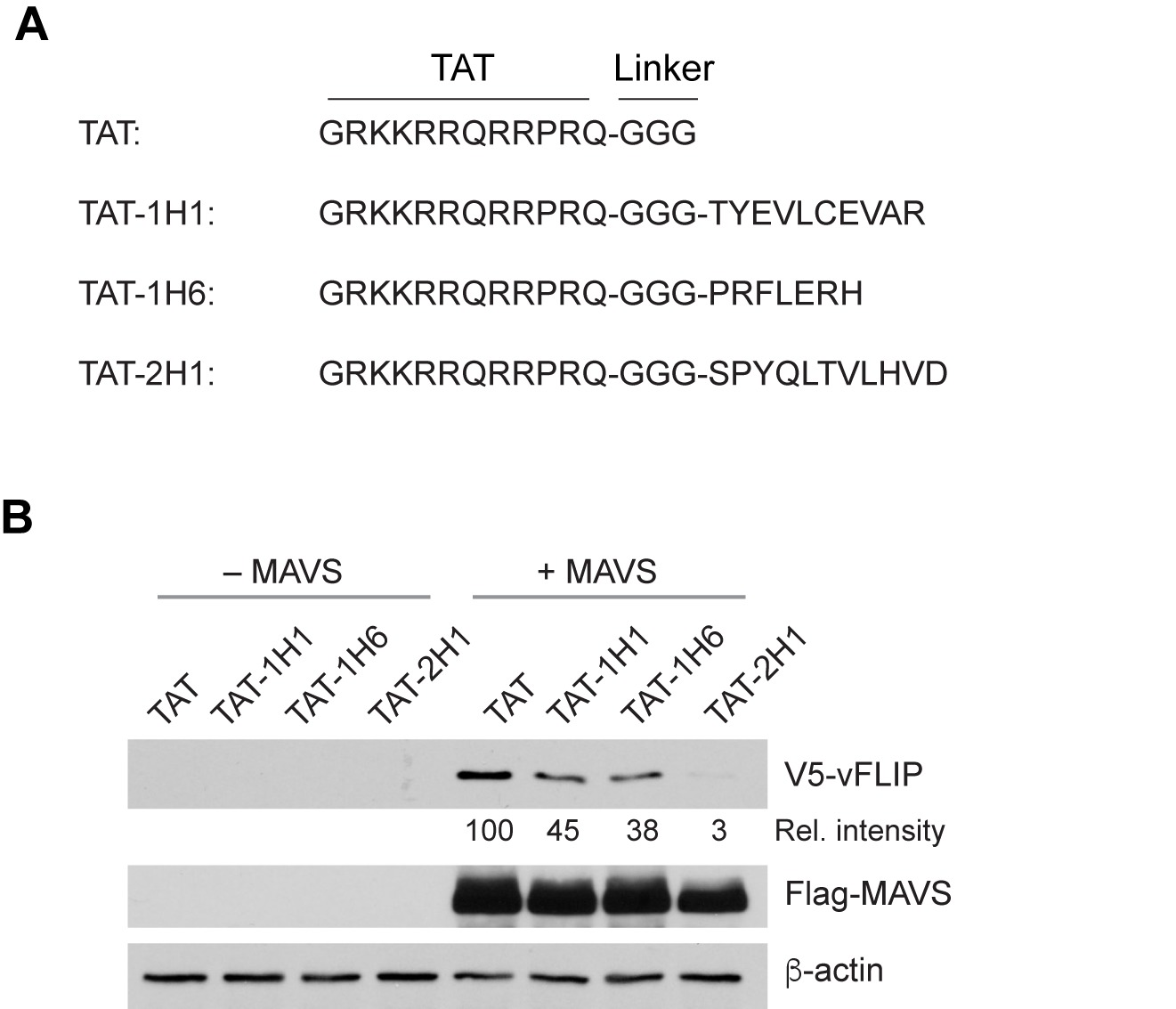

Supplement: S8 Fig — (A) Sequences of TAT and TAT-fused vFLIP peptides. (B) Immunoblotting with extracts of 293A cells co-transfected with V5-vFLIP and empty (–MAVS) or Flag-MAVS (+ MAVS) vectors and then treated with the peptides for 1 day. (TIF) [file ppat.1007058.s008.tif]

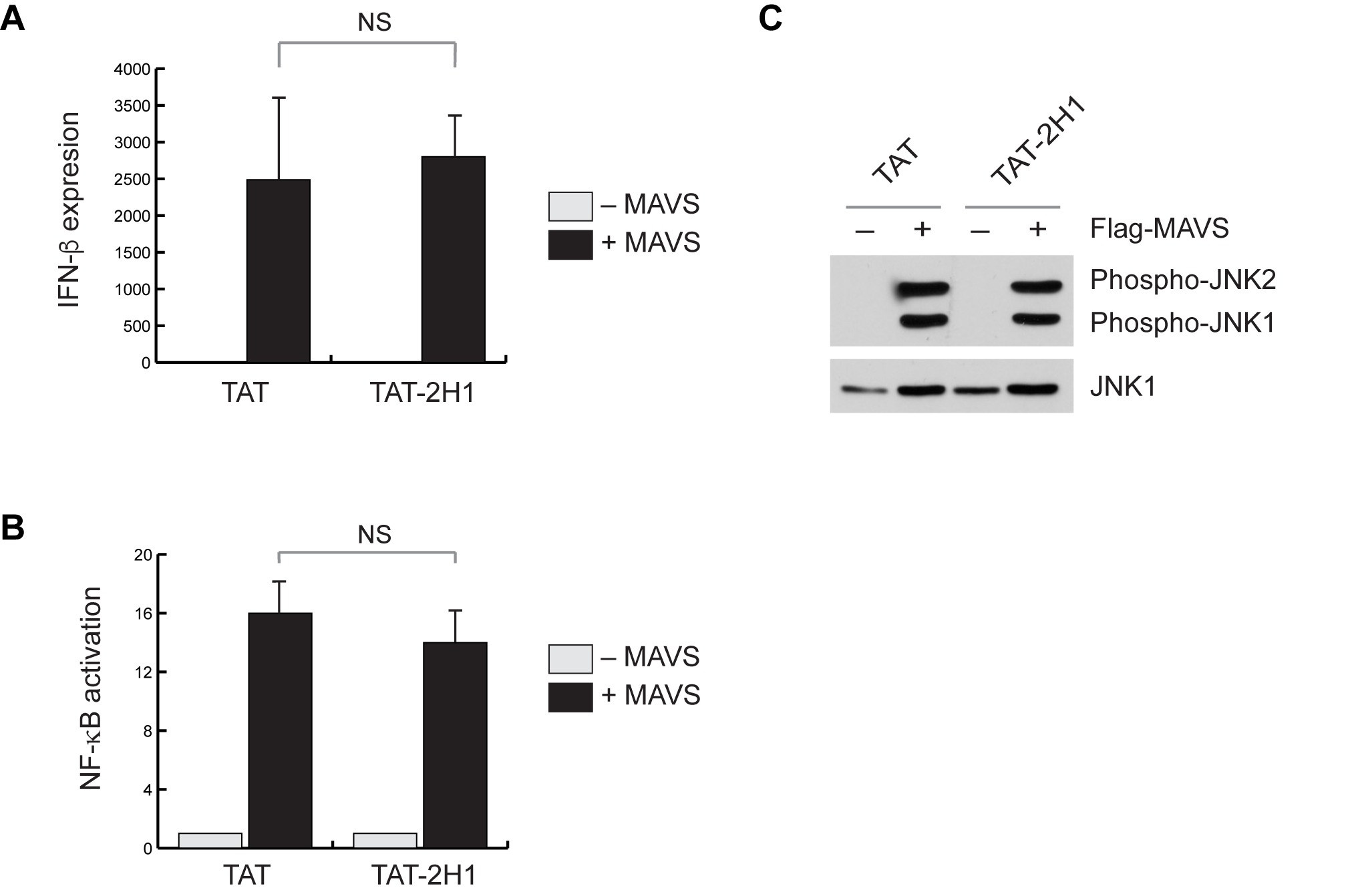

Supplement: S9 Fig — (A-B) Reporter assays in 293T cells transfected with empty (–MAVS) or Flag-MAVS (+ MAVS) vectors along with IFN-β-Luc (A) or NF-κB-Luc (B) reporter in the presence of TAT and TAT-2H1 peptides for 1 day. Data are presented as mean ± SD of triplicate samples. “NS” indicates not significant. (C) Immunoblots of extracts of 293T cells transfected with empty vector or MAVS plasmid in the presence of TAT and TAT-2H1 peptides for 1 day. (TIF) [file ppat.1007058.s009.tif]

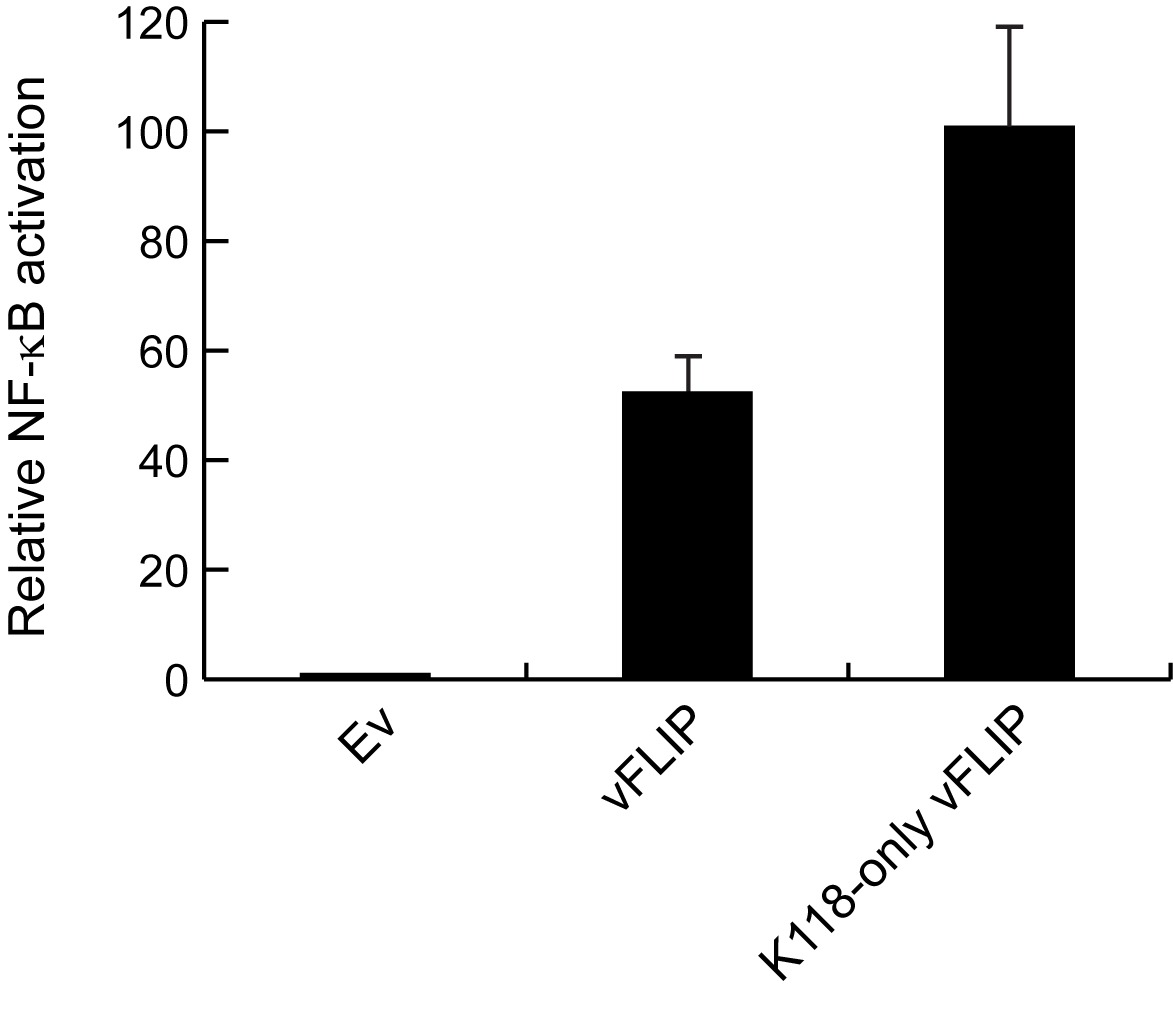

Supplement: S10 Fig — Reporter assay was performed in 293T cells transfected with empty vector, vFLIP, or K118-only vFLIP vectors along with NF-κB-Luc (B) reporter plasmid for 1 day. Data are presented as mean ± SD of triplicate samples. (TIF) [file ppat.1007058.s010.tif]

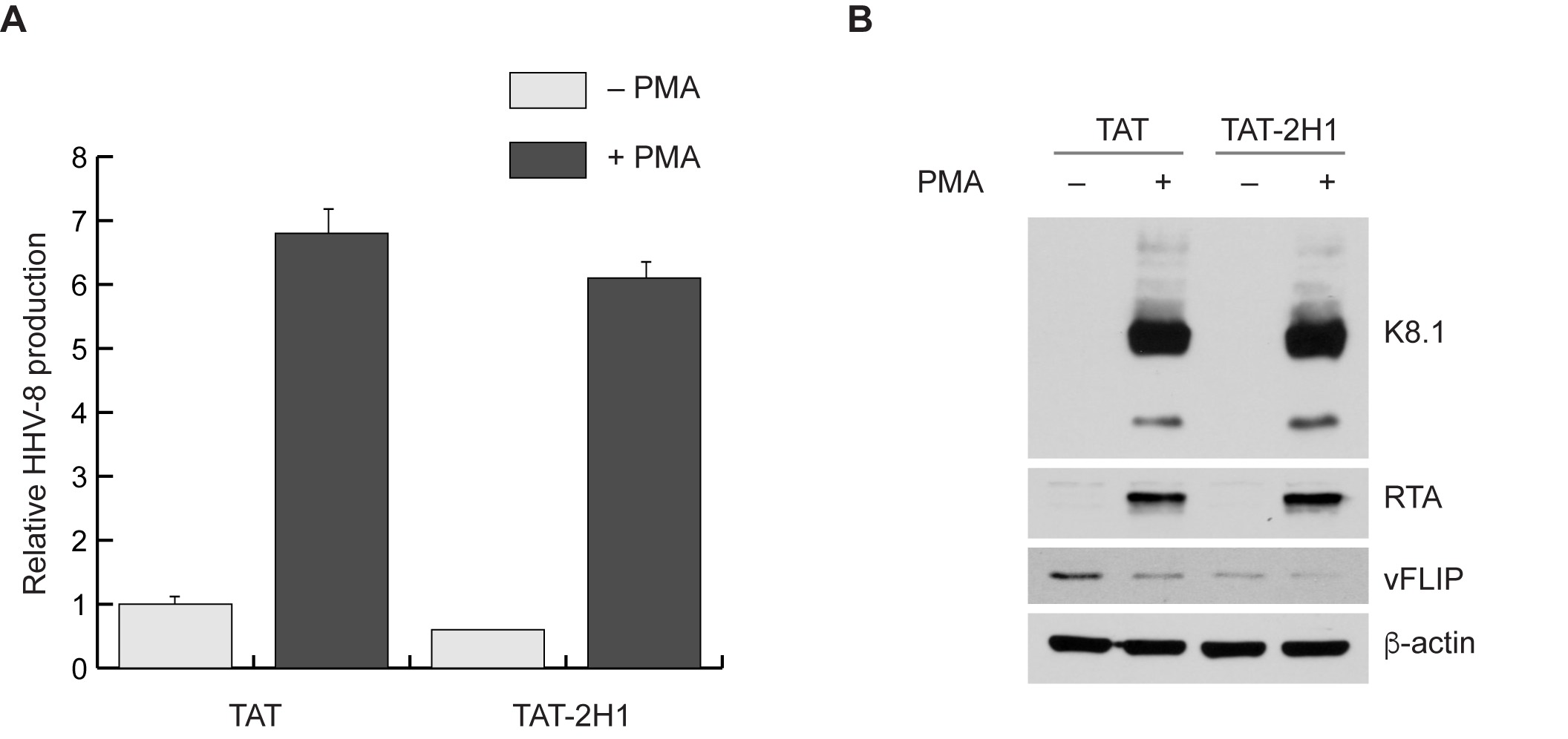

Supplement: S11 Fig — (A) RT-qPCR analysis of encapsidated HHV-8 genome in the supernatants of BCBL-1 culture. Cells were left untreated or treated with 20 ng/ml of phorbol myristate acetate (PMA), a lytic inducer, together with 10 μM TAT or TAT-2H1 peptides for 3 days. The viral genome copy number was determined using a standard curve of BAC16 DNA; fold change (relative copy number) was calculated by dividing the values of the samples with that of control (TAT and no PMA). Data are presented as mean ± SD of triplicate samples. (B) Immunoblotting analysis of extracts of the BCBL-1 cells treated as in (A). (TIF) [file ppat.1007058.s011.tif]
