# Supplementary material for: Peroxisomes support human herpesvirus 8 latency by stabilizing the viral oncogenic protein vFLIP via the MAVS-TRAF complex
Source: PLoS Pathog. 2018 May 10;14(5):e1007058. doi: 10.1371/journal.ppat.1007058 (PMC5963799; doi:10.1371/journal.ppat.1007058)
Supplement: S1 Table — (PDF) [file ppat.1007058.s012.pdf]

S1 Table. Oligonucleotides used in the study

Name	Forward or Reverse	Sequences (5' to 3')
MAVS gRNA1	Forward	CACCGTGGTCTCTTGCTGTGAGGCA
	Reverse	AAACTGCCTCACAGCAAGAGACCAC
MAVS gRNA3	Forward	CACCGCCTTCAGCGGCGGCCCGGCT
	Reverse	AAACAGCCGGGCCGCCGCTGAAGGC
PEX19 gRNA	Forward	CACCGTGAGGAAGGCTGTAGTGTCG
	Reverse	AAACCGACACTACAGCCTTCCTCAC
$MAVS^{Rg}$	Forward	ATTCTGCCTTACCTGCCCTGTCTGACTGCTAGGGATCAGGATCGACTGCGGGCCACC
	Reverse	GGTGGCCCGCAGTCGATCCTGATCCCTAGCAGTCAGACAGGGCAGGTAAGGCAGAAT
vFLIP	Forward	ACGAGGTTCTCTGTGAGGTG
	Reverse	AGACATTCCGCTAACAGGGG
IFN-α1	Forward	GCCTCGCCCTTTGCTTTACT
	Reverse	CTGTGGGTCTCAGGGAGATCA
IFN-β1	Forward	GCTTGGATTCCTACAAAGAAGCA
	Reverse	ATAGATGGTCAATGCGGCGTC
IFN-β2	Forward	ACTCACCTCTTCAGAACGAATTG
	Reverse	CCATCTTTGGAAGGTTCAGGTTG
IFN-γ	Forward	TCGGTAACTGACTTGAATGTCCA
	Reverse	TCGCTTCCCTGTTTTAGCTGC
IFN-λ1	Forward	CACATTGGCAGGTTCAAATCTCT
	Reverse	CCAGCGGACTCCTTTTTGG
MIR-2	Forward	ACAAGGACCGTCAATTCGATG
	Reverse	TGCCATACCGACGGCC
K8.1	Forward	TGCCATTTTCTGCCACCACTACAACGACT
	Reverse	ACAAGTCCCAGCAATAAACCCACAGCCCA
LANA	Forward	TACGGTTGGCGAAGTCACATC
	Reverse	CCTCGCAGCAGACTACACCTCCAC
β-actin	Forward	TGCCATCCTAAAAGCCACCCCACTTC
	Reverse	AAGCAATGCTATCACCTCCCCTGTGT
