# Supplementary material for: Peroxisomes support human herpesvirus 8 latency by stabilizing the viral oncogenic protein vFLIP via the MAVS-TRAF complex
Source: PLoS Pathog. 2018 May 10;14(5):e1007058. doi: 10.1371/journal.ppat.1007058 (PMC5963799; doi:10.1371/journal.ppat.1007058)
Supplement: S2 Table — (PDF) [file ppat.1007058.s013.pdf]

S2 Table. **Plasmids used in the study**

Name	SOURCE/Cloning or Mutagenesis	IDENTIFIER
lentiCRISPR V2-Puro	(Sanjana et al., 2014)	Addgene 52961
lentiCRISPR V2-Puro_gRNAs	Cloning using BsmBI	N/A
MAVS-Mito	(Dixit et al., 2010)	Addgene 44556
MAVS-Pex	(Dixit et al., 2010)	Addgene 44557
pICE	(Britton et al., 2013)	Addgene 46960
pICE_V5	Cloning using HindIII and NotI	N/A
pICE_V5-vFLIP	Cloning using BamHI and MluI	N/A
pICE_V5-vFLIP point mutants	Site-directed mutagenesis	N/A
pICE_V5-vFLIP-Pex	Cloning by overlap extension PCR	N/A
pICE_vFLIP (no tag)	Cloning using BamHI and MluI	N/A
pICE_V5-cFLIP-L and S	Cloning using BglII and MluI	N/A
pICE_GST	Cloning using HindIII and NotI	N/A
pICE_GST-vFLIP and derivatives	Cloning using EcoRI and XhoI	N/A
pFlag_MAVS and deletion mutants	(Hwang and Choi, 2016)	N/A
pFlag_MAVS point mutants	Site-directed mutagenesis	N/A
TRAF plasmids	(Choi and Harhaj, 2014)	N/A
HA-TRAF3 C68A/H70A	Site-directed mutagenesis	N/A
pICE_HA	Cloning using HindIII and NotI	N/A
pICE_HA-PEX19	Cloning using NotI and MluI	N/A
pICE_Flag	Cloning using HindIII and BamHI	N/A
pICE_Flag-MAVS	Cloning using BamHI and MluI	N/A
pICE_Flag-MAVS-Mito	Cloning using BamHI and MluI	N/A
pICE_Flag-MAVS-Pex	Cloning using BamHI and MluI	N/A
Myc-IKKγ	(Xiao and Sun, 2000)	N/A
pGEX 4T-1	GE Life Sciences	28-9545-49
pGEX 4T-1_vFLIP	Cloning using BamHI and XhoI	N/A
pGEX 4T-1_vFLIP mPTS^{X}	Site-directed mutagenesis	N/A
pGEX 4T-1_cFLIP-S	Cloning using BglII and XhoI	N/A
pGEX 4T-1_TRAF6	(Choi and Harhaj, 2014)	N/A
pcDNA3_HA-MC159	(Huttmann et al., 2015)	N/A
pICE_V5-MC159	Cloning using BamHI and MluI	N/A
pVenus C1_RRV vFLIP (ORF71)	(Ritthipichai et al., 2012)	N/A
pICE_V5-RRV vFLIP (ORF71)	Cloning using BamHI and MluI	N/A
pDUET011	(Zhou et al., 2007)	Addgene 17627
pDUET011_MAVS^{Rg1} and point mutants	Cloning using BglII and SalI	N/A
pDUET011_MAVS^{Rg1}-Pex	Cloning using BglII and SalI	N/A
pDUET011_K118-only vFLIP	Cloning using BamHI and SalI	N/A
psPAX2	Didier Trono	Addgene 12260
NF-κB-Luc	Stratagene	N/A
IFN-β-Luc	(Hwang and Choi, 2016)	N/A
pRL-TK	Promega	E2231
