# Supplementary material for: Peroxisomes support human herpesvirus 8 latency by stabilizing the viral oncogenic protein vFLIP via the MAVS-TRAF complex
Source: PLoS Pathog. 2018 May 10;14(5):e1007058. doi: 10.1371/journal.ppat.1007058 (PMC5963799; doi:10.1371/journal.ppat.1007058)
Supplement: S3 Table — (PDF) [file ppat.1007058.s014.pdf]

S3 Table. **Antibodies used in the study**

REAGENT or RESOURCE	SOURCE	IDENTIFIER
Mouse monoclonal anti-MAVS (E-3)	Santa Cruz Biotechnology	sc-166583
Mouse monoclonal anti-GST (B-14)	Santa Cruz Biotechnology	sc-138
Mouse monoclonal anti-TOM20 (F-10)	Santa Cruz Biotechnology	sc-17764
Mouse monoclonal anti-IKK gamma (B-3)	Santa Cruz Biotechnology	sc-8032
Mouse monoclonal anti-JNK1 (F-3)	Santa Cruz Biotechnology	sc-1648
Mouse monoclonal anti-K8.1A/B	Santa Cruz Biotechnology	sc-65446
Rabbit polyclonal anti-GFP (FL)	Santa Cruz Biotechnology	sc-8334
Rabbit polyclonal anti-LDH (H-160)	Santa Cruz Biotechnology	sc-33781
Rabbit polyclonal anti-His-probe (H-15)	Santa Cruz Biotechnology	sc-803
Mouse monoclonal anti-V5 tag	Thermo Fisher Scientific	R960-25
Mouse monoclonal anti-beta ACTIN (AC-15)	Abcam	ab6276
Rabbit monoclonal anti-K63-linkage polyUb	Abcam	ab179434
Rabbit polyclonal anti-LC3B	Novus Biologicals	NB100-2220
Rabbit polyclonal anti-PEX19	Novus Biologicals	NBP1-32925
Mouse monoclonal anti-PMP70	Sigma	SAB4200181
Mouse monoclonal anti-Flag tag (M2)	Sigma	F3165
Mouse monoclonal anti-V5-agarose	Sigma	A7345
Rat polyclonal anti-DYKDDDDK tag (L5) gel	BioLegend	651502
Rabbit polyclonal anti-p62 (SQSTM1)	MBL	PM045
Rabbit monoclonal anti-K48-linkage polyUb (D9D5)	Cell Signaling Technology	#8081
Rabbit polyclonal anti-Myc tag	Cell Signaling Technology	#2272
Rabbit polyclonal anti-DYKDDDDK tag	Cell Signaling Technology	#2368
Rabbit polyclonal anti-V5 tag (D3H8Q)	Cell Signaling Technology	#13202
Rabbit monoclonal anti-GST	Cell Signaling Technology	#2625
Rabbit polyclonal anti-phospho-SAPK/JNK	Cell Signaling Technology	#9251
Mouse monoclonal anti-HA (12CA5)	Roche	11583816001
Rabbit polyclonal anti-vFLIP	(Chmura et al., 2017)	N/A
Rabbit polyclonal anti-RTA	Dr. John Nicholas	N/A
Alexa Fluor488 goat anti-mouse IgG (H+L)	Thermo Fisher Scientific	A11029
Alexa Fluor594 goat anti-rabbit IgG (H+L)	Thermo Fisher Scientific	A11037
